# Novel Ferrocene Derivatives Induce Apoptosis through Mitochondria-Dependent and Cell Cycle Arrest via PI3K/Akt/mTOR Signaling Pathway in T Cell Acute Lymphoblastic Leukemia

**DOI:** 10.3390/cancers13184677

**Published:** 2021-09-18

**Authors:** Liao Zeng, Mingqing Tang, Chao Pi, Jianrong Zheng, Sanxing Gao, Titaua Chabanne, Remi Chauvin, Wenzhao Cheng, Hongjun Lin, Ruian Xu, Xiuling Cui

**Affiliations:** 1 Engineering Research Centre of Molecular Medicine of Ministry of Education, Fujian Key Laboratory of Molecular Medicine, Key Laboratory of Precision Medicine and Molecular Diagnosis of Fujian Universities, Xiamen Key Laboratory of Marine and Gene Drugs, School of Medicine, Huaqiao University, Xiamen 361021, China; 18014071002@stu.hqu.edu.cn (L.Z.); Tmq@hqu.edu.cn (M.T.); 18014071027@stu.hqu.edu.cn (J.Z.); 1511316017@stu.hqu.edu.cn (S.G.); titaua.chabanne@gmail.com (T.C.); chauvin@lcc-toulouse.fr (R.C.); 1300116002@stu.hqu.edu.cn (W.C.); 19014071011@stu.hqu.edu.cn (H.L.); ruianxu@hqu.edu.cn (R.X.); 2 Henan Key Laboratory of Chemical Biology and Organic Chemistry, Key Laboratory of Applied Chemistry of Henan Universities, Green Catalysis Center, and College of Chemistry, Zhengzhou University, Zhengzhou 450052, China; pichao@zzu.edu.cn; 3 LCC-CNRS, Université de Toulouse, UPS, 205 Route de Narbonne, 31077 Toulouse, France

**Keywords:** ferrocene derivatives, T cell acute lymphoblastic leukemia, apoptosis, reactive oxygen species, mitochondrial membrane potential, cell cycle, PI3K/Akt/mTOR signaling pathway

## Abstract

**Simple Summary:**

T cell acute lymphoblastic leukemia (T-ALL) is a malignant hematologic disease that urgently requires efficient therapeutic agents. The aim of this study is to explore the anti-T-ALL activity of novel ferrocene derivatives. It was found that ferrocene derivatives F1–F7 synthesized by our group inhibited the proliferation of several cancer cell lines in vitro. Among them, F1 and F3 displayed potent cytotoxicity against T-ALL cell lines, especially Jurkat cells, with low cytotoxicity for normal cells. Mechanistically, F1 and F3 could induce apoptosis through mitochondria-dependent pathway mediated by ROS, and cell cycle arrest at G0/G1 phase via the PI3K/Akt/mTOR signaling pathway in Jurkat cells. These results suggested that F1 and F3 could be potential candidates for future T-ALL therapy.

**Abstract:**

T cell acute lymphoblastic leukemia (T-ALL) is one of the most common causes of death in pediatric malignancies. However, the clinical chemotherapy for T-ALL has been limited by numerous side effects, emphasizing that novel anti-T-ALL drugs are urgently needed. Herein, a series of 2-acyl-1-dimethylaminomethyl-ferrocenes for cancer therapy have been evaluated. Among them, F1 and F3 exhibited potent cytotoxicity against T-ALL cell lines, especially Jurkat cells, with low cytotoxicity for normal cells. Further mechanistic studies revealed that F1 and F3 could induce apoptosis in Jurkat cells by destructing mitochondrial membrane, enhancing reactive oxygen species (ROS) generation, decreasing the Bcl-2/Bax ratio, releasing Cytochrome c, and increasing the expression of Cleaved Caspase-9/-3 and Cleaved PARP. Additionally, F1 and F3 could suppress cell proliferation and arrest the cell cycle at G0/G1 phase through the PI3K/Akt/mTOR signaling pathway by down-regulating the expression of CDK6, Cyclin D1, p-Akt, p-GSK-3β, p-mTOR, p-p70 S6K, and up-regulating the expression of P21 and P27, which would also be a possible mechanism. Consequently, ferrocene derivatives F1 and F3 could induce apoptosis through a mitochondria-dependent pathway mediated by ROS, and cell cycle arrest at G0/G1 phase via the PI3K/Akt/mTOR signaling pathway in Jurkat cells. The present study provided fundamental insights into the clinical application of F1 and F3 for the treatment of T-ALL.

## 1. Introduction

Leukemia is a cancer of the blood cells and starts in blood-forming tissue, such as the bone marrow. American Cancer Society (ACS) estimated that there are approximately 61,090 new cases of leukemia in 2021 [1]. The types of leukemia are grouped by how quickly the disease develops and gets worse (chronic vs. acute), or by which blood cells are affected (lymphoid vs. myeloid) [2]. For example, adult T cell leukemia-lymphoma (ATL) is one type of chronic leukemia and a distinct mature T cell malignancy caused by chronic infection with human T-lymphotropic virus type 1 (HTLV-1). Recently, great progress has been made in this field. We found that the aryl hydrocarbon receptor (AHR) was a tunable knob and controlled HTLV-1 latency-reactivation switching [3], and the activation of Notch1 signaling by HTLV-1 Tax promoted proliferation of adult T cell leukemia cells [4]. T cell acute lymphoblastic leukemia (T-ALL), a malignant hematologic disease characterized by the uncontrolled proliferation of immature T-lymphoid cells, is more frequently reported in pediatric patients and hard to cure [5]. The clinical treatment for T-ALL patients includes chemotherapy, radiation therapy, stem cell transplantation, and targeted therapy. Despite the improvement of cure rate, 15–20% of patients relapse and the mortality rate is as high as 50–70% [6,7]. Chemotherapeutic agents, such as Vincristine, Daunorubicin and Prednisone, are currently used as one of the dominant clinical regimens. Unfortunately, they are limited by high toxicity and drug resistance [8,9]. Based on that, developing novel anti-T-ALL drugs with a safe profile is highly desired.

On the other hand, ferrocene has gained increasing interest of medicinal chemists due to its sandwich structure and unique chem- and bio-properties, such as lipophilic, low toxicity, air- and water-stable [10,11,12]. For instance, ferrocifen represents a promising candidate for anti-cancer treatments and appears to be highly selective to breast cancer cells [13,14]. Moreover, N^1^-Hydroxy-N^8^-ferrocenyloctanediamide (JAHA), an organometallic analog of suberoylanilide hydroxamic acid (SAHA), displays inhibition of class I histone deacetylase (HDACs), excellent selectivity over class IIa HDACs, and anti-cancer action in cells [15]. Our group reported that ferrocenyl olefins exhibited a broad anti-cancer spectrum [16]. Most recently, we demonstrated that 2-acyl-1-dimethylaminomethyl-ferrocenes selectively inhibited the proliferation of hepatocellular carcinoma cell lines through a mitochondrial pathway [17].

In continuing our interest in the bioactivities of ferrocene derivatives, we embarked upon the investigation on the anti-cancer potential of 2-acyl-1-dimethylaminomethyl-ferrocenes. The results obtained revealed that both ferrocene derivatives F1 and F3 inhibited the proliferation of Jurkat cells with relatively mild toxicity for normal cells. Further mechanistic studies firstly suggested that F1 and F3 could induce apoptosis through a mitochondria-dependent pathway mediated by ROS and arrest the cell cycle at G0/G1 phase via the PI3K/Akt/mTOR signaling pathway in Jurkat cells.

## 2. Materials and Methods

### 2.1. Synthesis of F1–F7

Compounds F1–F7 were synthesized according to the procedure developed by our group [18]. All the compounds were dissolved in dimethyl sulfoxide (DMSO) (Beyotime, Shanghai, China). The concentration of DMSO never exceeded 0.1% (v/v). An amount of 50 mM of stock solution was stored at −20 °C. All other chemicals used were commercially available and reagent grade.

### 2.2. Cell Cultures

Jurkat (Human acute T lymphoma leukemia cell line), Molt-4 (Human acute T lymphoblastic leukemia cell line), CEM-T4 (Human acute T lymphoma leukemia cell line) used in this experiment were obtained from Professor Masao Matsuoka (Kumamoto University, Japan) and cultured in RPMI 1640 medium (Gibco, Grand Island, NY, USA). RAJI and CA46 (Human Burkitt lymphoma cell line), SNT8 and SNK6 (Human NK/T lymphoma cell line) were purchased from Shanghai Institute of Cell Biology and cultured in RPMI 1640 medium (Gibco, Grand Island, NY, USA). HEK293 (Human embryonic kidney cell line) was purchased from the Shanghai Institute of Cell Biology and grown in DMEM medium (Gibco, Grand Island, NY, USA). All of them were supplemented with 10% fetal bovine serum (FBS) (Gibco, Grand Island, NY, USA), 100 U/mL penicillin and 100 µg/mL streptomycin (Solarbio, Beijing, China) at 37 °C in the presence of 5% CO_2_.

### 2.3. Isolation and Culture of Human Peripheral Normal T Cells

Human peripheral blood mononuclear cells (PBMC) were separated by Ficoll Lymphocyte Separation Solution (Solarbio, Beijing, China) according to its instructions. Briefly, the heparinized whole blood sample obtained from the healthy volunteer was carefully poured over the lymphocyte separation solution. The separation process was performed by centrifugation at 1000 g for 30 min over Allegra X-15R Centrifuge (Beckman Coulter, Indianapolis, IN, USA). The lymphocytes concentrated in the interface (white layer) were extracted carefully and washed twice in culture medium. Subsequently, the isolation of normal human T cells from PBMC was performed using EasySep™ HLA T Cell Enrichment Kit (STEMCELL Technologies, Vancouver, BC, Canada). Phytohemag-glutinin A (PHA) (Solarbio, Beijing, China) was used to activate the normal human T cells at a concentration of 4% for 60 h. This protocol was approved by the Ethics Committee of Huaqiao University, Quanzhou, China (No. M2021012). Additionally, informed consent was obtained from the volunteer.

### 2.4. Cell Viability Analysis

Cell viability was measured by CCK-8 Assay Kit (Beyotime, Shanghai, China). Cells were seeded in 96-well plates at a density of 5 × 10^3^ cells/well. Twelve hours later, the final concentrations of F1–F7 were adjusted to 6.25, 12.5, 25, 50, 100 µM, and Daunorubicin (Solarbio, Beijing, China) was adjusted to 0.0625, 0.125, 0.25, 0.5, 1 µM, and added into 96-well plate. The 0 μM group was set as the control group. Cell viability was measured after 48 h. The CCK-8 (20 μL) was added to each well and incubated for 2 h in the dark at 37 °C. The absorbance of the solution was measured at 450 nm using Varioskan LUX (Thermo Fisher Scientific, Waltham, MA, USA). Cell viability was calculated based on the following formula: Viability = OD450 (treated group)/OD450 (control group). The IC_50_ values were expressed as inhibited cell growth by 50%, analyzed by Graph Prism 7.0 (Graph Pad Saftware, La Jolla, CA, USA).

### 2.5. Hoechst 33258 Fluorescent Staining

Cells were seeded in 96-well plates at a density of 5 × 10^3^ cells/well. After 12 h, cells were treated with serial concentrations of F1 and F3 for 48 h and then harvested to stain with Hoechst 33258 (Beyotime, Shanghai, China). Cells were collected by centrifugation under 500× *g* × 5 min, followed by fixing with 4% paraformaldehyde (Solarbio, Beijing, China) for 10 min. Then, the cells were incubated with Hoechst 33258 for 15 min at room temperature, followed by centrifugation and rinsing with phosphate buffer saline (PBS) (Beyotime, Shanghai, China) twice, resuspended in PBS and transferred to new 96-well plates. Subsequently, the images of nuclear-related apoptosis were captured with the NIS-Elements Image Software (Version 4.0) (Nikon, Tokyo, Japan).

### 2.6. Cell Cycle Analysis

Jurkat cells were seeded in 6-well plates at a concentration of 2 × 10^5^ cells/well. After 12 h, cells were incubated with F1 or F3 for 48 h, respectively. Then, the cells were collected and washed with PBS twice, and subsequently fixed with 70% ethanol overnight at 4 °C. After fixation, cells were centrifuged and washed twice with PBS. To ensure that only DNA was being measured, the cells were resuspended in PBS containing 50 mg/mL RNase A (Sigma-Aldrich, St. Louis, MO, USA) at 37 °C for 30 min, followed by 1× PBS and 0.25 mg/mL PI (Sigma-Aldrich, St. Louis, MO, USA) in the dark. Next, the cells were centrifuged and then resuspended in 500 μL of 1× PBS. DNA content of cells was determined by FACSCalibur flow cytometer (BD Biosciences, San Jose, CA, USA) on CellQuest Pro software (BD Biosciences, San Jose, CA, USA) with default setting. Excitation wavelength Ex = 535 nm and emission wavelength Em = 617 nm.

### 2.7. Assessment of Apoptosis

Jurkat cells were seeded in 6-well plates at a concentration of 2 × 10^5^ cells/well. After 12 h, cells were treated with serial concentrations of F1 and F3. After 48 h incubation at 37 °C, cells were harvested and washed 3 times with cold PBS. Apoptosis was detected by an FITC Annexin V Apoptosis detection Kit (BD Biosciences, San Jose, CA, USA) according to the manufacturer’s protocol. Cells were centrifuged and then resuspended in 500 μL of 1× binding buffer. Then, the cells were stained with 5 μL Annexin V-FITC (BD Biosciences, San Jose, CA, USA) and 5 μL PI (BD Biosciences, San Jose, CA, USA) at 37 °C for 15 min in the dark. The fluorescence of the cells was immediately determined by FACSCalibur flow cytometer (BD Biosciences, San Jose, CA, USA). The percentages of apoptotic cells were analyzed by CellQuest Pro software (BD Biosciences, San Jose, CA, USA). Excitation wavelength Ex = 488 nm and emission wavelength FL1 (Em = 525 nm); FL2 (Em = 615 nm).

### 2.8. ROS Detection

The intracellular increase of ROS upon F1 and F3 was measured by ROS Assay Kit (Beyotime, Shanghai, China) according to the manufacturer’s protocol. Cells were seeded in 24-well plates at 1 × 10^5^ cells/well for 12 h, treated with F1 or F3 for 48 h at different concentrations, and then incubated with a cell-permeable fluorogenic probe 2′,7′-dichlorodihydrofluorescein diacetate (DCFH-DA) (final concentration = 10 µM) at 37 °C for 15 min. Then, the cells were rinsed and resuspended with FBS free culture medium 3 times and examined by the NIS-Elements Image Software (Version 4.0) (Nikon, Tokyo, Japan) built-in the ECLIPSE Ti fluorescence microscope (Nikon, Tokyo, Japan).

### 2.9. Mitochondrial Membrane Potential Analysis

Mitochondrial membrane potential (MMP) of Jurkat cells after treatment with F1 or F3 at 48 h was determined using a JC-1 Mitochondrial Membrane Potential Assay Kit (Beyotime, Shanghai, China) according to the manufacturer’s instructions. Briefly, Jurkat cells were seeded in 24-well plates at 1 × 10^5^ cells/well and treated with F1 or F3. After 48 h, 10 µL of JC-1 dyeing working solution was added into each well and incubated at 37 °C for 30 min. Later, cells were washed with JC-1 staining buffer (1×) 3 times, and then resuspended with JC-1 staining buffer (1×). Then, the samples were examined with FACSCalibur flow cytometer (BD Biosciences, San Jose, CA, USA) and analyzed with CellQuest Pro software (BD Biosciences, San Jose, CA, USA). Excitation wavelength Ex = 488 nm and emission wavelength FL1 (Em = 579 nm); FL2 (Em = 599 nm).

### 2.10. Western Blot Analysis

Jurkat cells were seeded in a 6-well plate. After 12 h, the cells were treated by F1 or F3 for 72 h. Cells were then collected, washed by PBS, and lysed by radio-immunoprecipitation assay (RIPA) buffer (Beyotime, Shanghai, China) containing Protease Inhibitor and Phosphatase Inhibitor (Thermo Fisher Scientific, Waltham, MA, USA). Lysates were centrifuged at 13,500× *g* for 10 min at 4 °C. Then, the protein concentration of the supernatant was detected using Bradford Protein Assay Kit (Beyotime, Shanghai, China) according to the standard procedure. After separated by SDS-PAGE on 10% or 15% polyacrylamide gels, the protein was transferred to polyvinylidene fluoride (PVDF) membrane (MiliporeSigma, Burlington, MA, USA). Subsequently, membranes were blocked in 5% skimmed milk (BD Biosciences, San Jose, CA, USA) at room temperature for 3 h, and then incubated with primary antibodies overnight at 4 °C. The primary antibodies anti-Bcl-2, Bax, Cyto c, Cleaved Caspase-9, Cleaved Caspase-3, PARP, CDK6, Cyclin D1, Akt, p-Akt (Ser473), p-Akt (Thr308), mTOR, p-mTOR (Ser2448), p70 S6K, p-p70 S6K (Ser389) were purchased from Cell Signaling Technology (Beverly, MA, USA) and P21, P27, GSK-3β, p-GSK-3β (Ser9), β-tubulin, GAPDH were purchased from Beyotime (Shanghai, China). The membranes were washed 3 times with TBS-T (0.01%) and incubated with an appropriate horseradish peroxidase (HRP)-conjugated secondary antibody (Beyotime, Shanghai, China) for 1 h at room temperature. The protein bands were visualized with BeyoECL Star (Beyotime, Shanghai, China) after cleaning the membrane 3 times with TBS-T. Images were captured by the gel imaging system (version 4600SF) (Tanon, Shanghai, China). Then, the proteins level was analyzed with Image J software (NIH, Bethesda, MD, USA).

### 2.11. Statistical Analysis

All analyses were processed by Student’s *t*-test or analysis of variance (ANOVA) using GraphPad Prism 7.0 (Graph Pad Saftware, La Jolla, CA, USA). The data were provided as mean ± SEM. In each case, *p* < 0.05 was considered statistically significant.

## 3. Results

### 3.1. F1 and F3 Selectively Inhibited the Proliferation of Jurkat Cells

**Figure 1:**
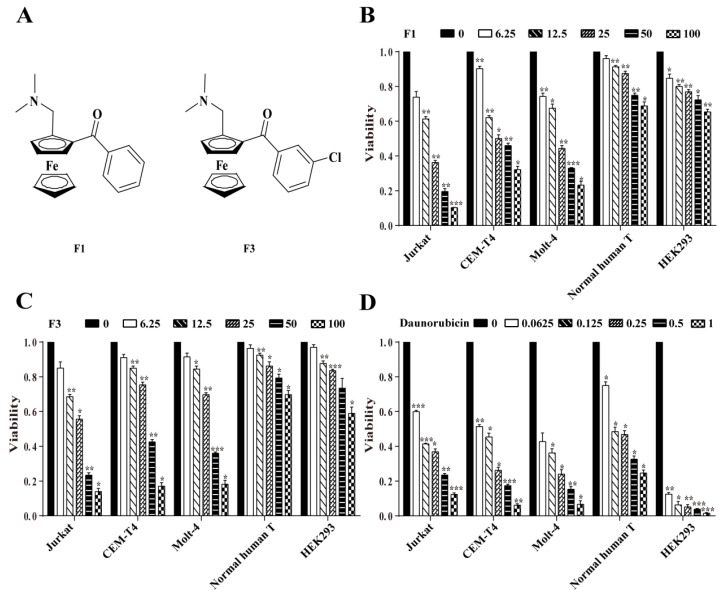
Viability of Jurkat, CEM-T4, Molt-4, Normal human T, and HEK293 cells treated by F1, F3, and Daunorubicin, respectively. (**A**) Chemical structures of F1 and F3. (**B**–**D**) Viability of Jurkat, CEM-T4, Molt-4, Normal human T, and HEK293 after in the presence of F1, or F3 at different concentrations (0, 6.25, 12.5, 25, 50 and 100 µM) for 48 h. Daunorubicin at different concentrations (0.0625, 0.125, 0.25, 0.5 and 1 µM) for 48 h. The data were expressed as the mean ± SEM of three independent experiments. * *p* < 0.05, ** *p* < 0.01 and *** *p* < 0.001 vs. the control group.

To evaluate the potential anti-cancer activity of compounds F1–F7 which were synthesized according to the procedure previously developed in our group (Figure S1) [18], the inhibition rates of F1–F7 against six cancer cell lines (Jurkat, CEM-T4, RAJI, CA46, SNT8, and SNK6) were detected by CCK8 assay. The preliminary screening results showed that F1–F7 all displayed cytotoxicity on cancer cell lines, while F1 and F3 were the most active compounds against T-ALL cell lines (Figure S2). Therefore, F1 and F3 were selected for further anti-T-ALL investigation. As shown in Figure 1, F1 and F3 inhibited the viability of T-ALL cell lines (Jurkat, CEM-T4, and Molt-4) in a dose-dependent manner, whereas exhibited relatively low cytotoxicity against normal cells (normal human T and HEK293) when compared with Daunorubicin (the positive control). Furthermore, the half-maximal inhibitory concentration (IC_50_) values in Table S1 indicated that F1 and F3 had much higher anti-cancer activity in Jurkat cells. Moreover, our previous study indicated that F1 could induce G0/G1 cell cycle arrest and apoptosis through the mitochondrial pathway in several human hepatocellular carcinoma cell lines [17]. Combined with the fact that the Jurkat cell line is extensively employed as a model of T cell signaling [19] and an appropriate model for drug investigation [20], the molecular mechanism of F1 and F3 in Jurkat cells was further studied.

### 3.2. F1 and F3 Changed the Cell Morphology and Induced Apoptosis in Jurkat Cells

**Figure 2:**
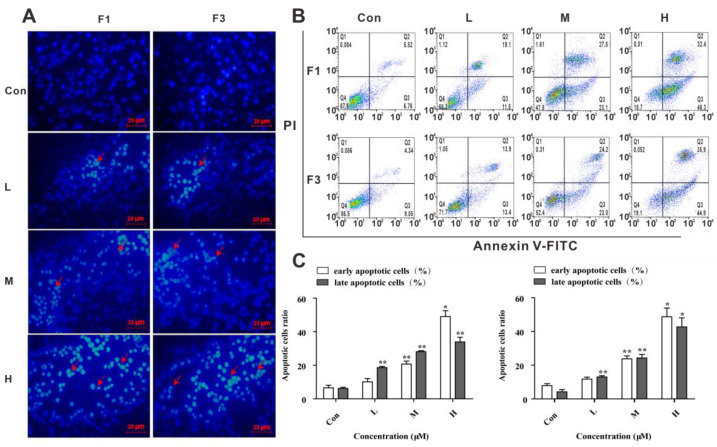
F1 and F3 induced apoptosis of Jurkat cells. (**A**) The morphological changes in Jurkat cells treated with F1 (F1-L: 10 µM, F1-M: 20 µM, F1-H: 40 µM) or F3 (F3-L: 15 µM, F3-M: 30 µM, F3-H: 50 µM) were determined by Hoechst 33258 staining (Scale bar =25 µm, 400×). (**B**) Effects of F1 or F3 at different concentrations for 48 h on the apoptosis of Jurkat cells were detected by the flow cytometer. (**C**) The sum of early and late apoptotic cells ratio was calculated. The data were shown as mean ± SEM of three independent experiments; * *p* < 0.05 and ** *p* < 0.01 vs. the control group.

In an attempt to observe the influence of F1 and F3 on morphology, Jurkat cells were stained by Hoechst 33258. Images from the fluorescence microscope showed that the cell morphology was largely changed with chromatin condensation and fragmentation compared with the control group in the presence of F1 or F3, indicating that apoptosis was induced by F1 or F3 in Jurkat cells (Figure 2A).

To further determine the extent of apoptosis in Jurkat cells treated by F1 or F3, Annexin V-FITC/PI double staining assay was performed. Compared with the control group, the percentage of early apoptotic cells and later apoptotic cells were significantly elevated from 12.28 ± 3.48% to 80.70 ± 3.13% when treated by 40 µM F1 for 48 h (Figure 2B,C). Meanwhile, a similar result was obtained from the Jurkat cells treated by F3. The above results further confirmed that F1 and F3 could inhibit the proliferation of Jurkat cells by inducing apoptosis in a dose-dependent manner.

### 3.3. F1 and F3 Increased Cellular ROS and Down-Regulated the MMP in Jurkat Cells

**Figure 3:**
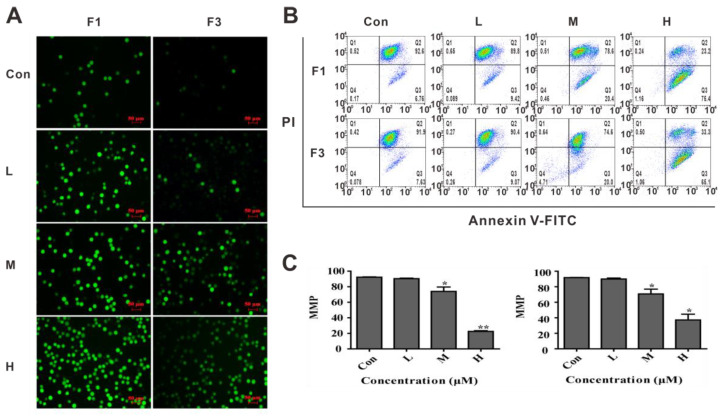
F1 and F3 induced ROS production and contributed to the MMP decrease in Jurkat cells. (**A**)The level of ROS in Jurkat cells was observed by fluorescence microscope after treated with F1 or F3 for 48 h at different concentrations (Scale bar =50 µm, 200×). (**B**,**C**) Changes in the MMP levels in Jurkat cells after treated with F1 or F3 at different concentrations were determined by flow cytometer. The shift-down of fluorescence from FL-2 (red fluorescence) to FL-1 (green fluorescence) indicated the collapse of MMP. Data were presented as mean ± SEM of three independent experiments; * *p* < 0.05 and ** *p* < 0.01 vs. the control group.

It is generally accepted that the production and accumulation of ROS would in turn ultimately lead to the damage of mitochondrial membrane and apoptosis [21,22]. Thus, the effects of F1 and F3 on the level of ROS were investigated by the DCFH-DA method. As shown in Figure 3A, the percentage of green fluorescence cells increased in a dose-dependent manner after treated with F1 and F3 for 48 h, respectively, which implied that F1 and F3 could increase the level of ROS in Jurkat cells.

To confirm whether apoptosis induced by F1 and F3 was related to the damage of mitochondrial membrane, the fluorescent probe JC-1 was used as an indicator of mitochondrial inner membrane depolarization to monitor the integrity of the mitochondrial membrane. As shown in Figure 3B,C, a significant decrease of MMP was observed by the flow cytometer in Jurkat cells treated with F1 or F3, which reflected the induction of early apoptosis. Taken all data above together, the changes in ROS and MMP were consistent with the results obtained above for the apoptosis assay.

### 3.4. F1 and F3 Regulated the Expression of Apoptosis-Related Proteins

**Figure 4:**
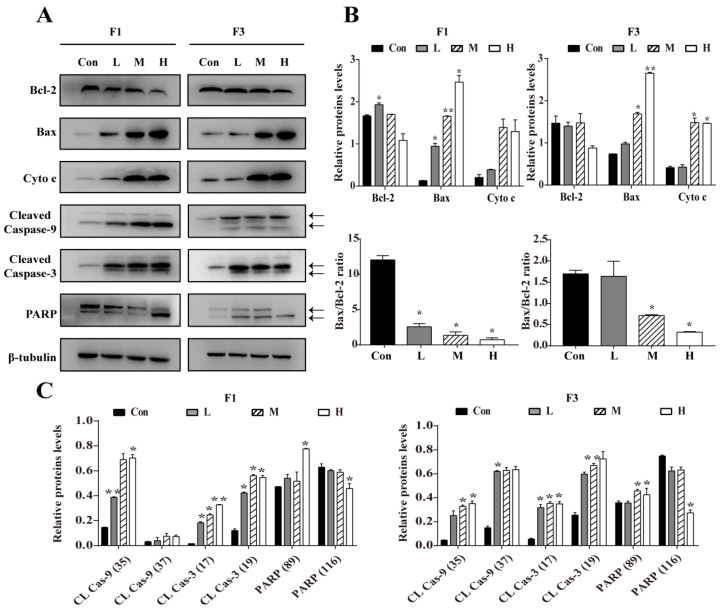
Apoptosis-associated proteins in Jurkat cells treated by F1 or F3 were analyzed by Western blot. (**A**) The expression of Bcl-2, Bax, Cytochrome c (Cyto c), Cleaved Caspase-9 (35/37 KD), Cleaved Caspase-3 (17/19 KD), and PARP (89/116 KD) in Jurkat cells treated by various concentrations of F1 or F3. β-tubulin was used as an internal control. (**B**) The histogram showed the relative protein levels and Bax/Bcl-2 ratio in Jurkat cells treated by F1 or F3. (**C**) The histogram showed the relative protein level in Jurkat cells treated by F1 or F3. Data were presented as mean ± SEM of three independent experiments; * *p* < 0.05 and ** *p* < 0.01 vs. the control group. The uncropped Western Blot images can be found in Figure S3.

To further explore the mechanism underlying the F1 and F3 induced apoptosis in Jurkat cells, the expression of apoptotic markers and anti-apoptotic factors were measured by Western blot analysis. As shown in Figure 4, the expression of Bcl-2 was significantly and dose-dependently decreased in Jurkat cells treated by F1 or F3. Meanwhile, the expression of apoptotic factors, such as Bax, Cytochrome c, Cleaved Caspase-9, Cleaved Caspase-3, and Cleaved PARP in Jurkat cells treated by F1 or F3 was increased. It is well known that the Bcl-2 family proteins are the key regulators of the mitochondrial pathway-mediated apoptosis [23], and the mitochondrial pathway of cell apoptosis involves the release of Cytochrome c and activation of Caspase-9 [24]. Therefore, the above findings not only confirmed that F1 and F3 induced apoptosis but also suggested that a mitochondria-dependent pathway might be involved.

### 3.5. F1 and F3 Induced Cell Cycle Arrest and Regulated the Expression of Cell Cycle-Related Proteins in Jurkat Cells

**Figure 5:**
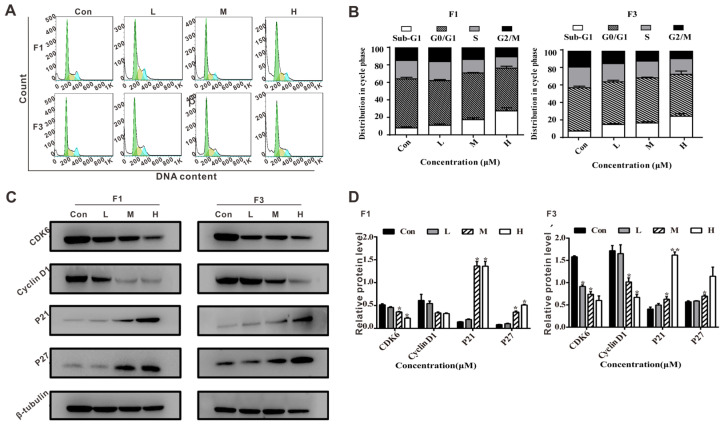
F1 and F3 triggered cell cycle arrest in Jurkat cells. (**A**) The cell cycle distribution of Jurkat cells treated with F1 or F3 at different concentrations (Sub-G1 phase, blank; G0/G1 phase, green; S phase, yellow; and G2/M phase, blue). (**B**) The histogram showed the distribution of cells (%) in Sub-G1, G0/G1, S, and G2/M phase. (**C**) The expression of CDK6, Cyclin D1, P27, and P21 in Jurkat cells treated by various concentrations of F1 or F3. β-tubulin was used as an internal control; (**D**) The histogram showed the relative protein levels in Jurkat cells treated by F1 or F3. Data were shown as mean ± SEM of three independent experiments; * *p* < 0.05 and ** *p* < 0.01 vs. the control group. The uncropped Western Blot images can be found in Figure S3.

To more extensively explore the molecular mechanism, the cytostatic effects of F1 or F3 on Jurkat cells were subjected to PI staining. The cell cycle distribution underwent significant changes in all treatment groups when compared with the control group. The proportion of Sub-G1 and G0/G1 phase cells was increased from 63.83 ± 3.09% to 73.23 ± 5.73%, and the percentage of G2/M phase cells was largely decreased from 14.61 ± 0.70% to 10.77 ± 3.13% in Jurkat cells treated by F1 (Figure 5A,B). Meanwhile, F3 showed a similar trend in Jurkat cells. Interestingly, it was observed that Jurkat cells treated with F1 and F3 resulted in the appearance of a Sub-G1 peak (apoptotic cells), and the peak increase was accompanied with an increase in concentrations, which reconfirmed the apoptosis assay data above. The changes in cell cycle distribution strongly suggested that F1 and F3 could induce cell cycle arrest at G0/G1 phase in a dose-dependent manner.

Following confirming that F1 and F3 could induce cell cycle arrest, the cell cycle-related proteins were assessed by Western blot. As shown in Figure 5C,D, the expression of CDK6 and Cyclin D1 were decreased while the expression of P27 and P21 were increased in the presence of F1, and a similar tendency had been found for F3. Thus, all evidence obtained from lab experiments suggested that F1 and F3 did induce apoptosis and cell cycle arrest in Jurkat cells.

### 3.6. F1 and F3 Suppressed the PI3K/Akt/mTOR Signaling Pathway

**Figure 6:**
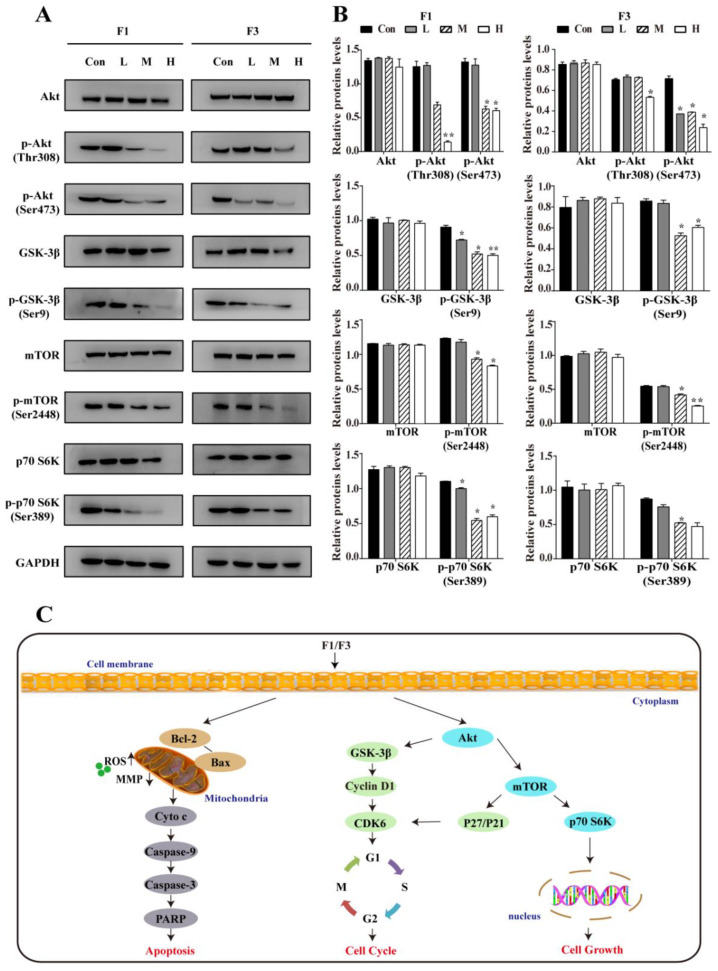
F1 and F3 suppressed the PI3K/Akt/mTOR signaling pathway in Jurkat cells. (**A**) The expression of Akt, p-Akt (Ser473), p-Akt (Thr308), GSK-3β, p-GSK-3β (Ser9), mTOR, p-mTOR (Ser2448), p70 S6K, p-p70 S6K (Ser389) in Jurkat cells treated by F1 or F3 were detected by Western blot. GAPDH was used as an internal control. (**B**) The histogram showed the relative protein levels in Jurkat cells treated by F1 or F3. Data were shown as mean ± SEM of three independent experiments; * *p* < 0.05 and ** *p* < 0.01 vs. the control group. (**C**) The possible mechanism of F1 and F3 effect in Jurkat cells. The pro-apoptotic effects of F1 or F3 were attributed to the activation of a mitochondria-dependent pathway mediated by ROS. On the other hand, the inhibition of the PI3K/Akt/mTOR signaling pathway could ultimately suppress cell growth and arrest cell cycle at G0/G1 phase. The uncropped Western Blot images can be found in Figure S3.

It is well known that the PI3K/Akt/mTOR signaling pathway is involved in cell growth, cell cycle regulation, and resistance to chemotherapy [25,26]. Since F1 and F3 could arrest the cell cycle at G0/G1 phase, the effects of F1 and F3 on the PI3K/Akt/mTOR signal pathway were further evaluated in Jurkat cells. As shown in Figure 6A,B, both F1 and F3 appeared to decrease the expression of p-Akt (Ser473), p-Akt (Thr308), p-GSK-3β (Ser9), p-mTOR (Ser2448), p-p70 S6K (Ser389) dose-dependently. However, the expression of corresponding total proteins had no significant changes. Altogether, F1 and F3 could inhibit the proliferation and cell cycle progression of Jurkat cells by suppressing the PI3K/Akt/mTOR signaling pathway.

## 4. Discussion

Although conventional chemotherapy plays an important role in the clinical treatment of T-ALL, serious problems remain, such as toxicity and drug resistance. Therefore, the development of potent and selective anti-T-ALL drugs is urgently needed. In the past decades, there has been increasing interest in ferrocene derivatives, especially for their anti-cancer activities. Recently, a series of 2-acyl-1-dimethylaminomethyl-ferrocenes F1–F7 have been synthesized by our group [18], and we reported that F6 selectively inhibited the proliferation of hepatocellular carcinoma cell lines through a mitochondrial pathway [17]. Herein, to continuing our interest in the bioactivity of F1–F7, their anti-cancer activities were well evaluated. The current results are summarized and shown in Figure 6C. All these data displayed that F1 and F3 were of high activity for anti-T-ALL but had relatively mild toxicity for normal cells (normal human T and HEK 293). It is noteworthy that when compared with F1, F3 had less anti-T-ALL activity probably due to its electron-attracting substituent which may therefore decrease biological activity and binding affinity of the benzoyl group [27]. Furthermore, F1 and F3 impaired the viability of Jurkat cells by inducing apoptosis through a mitochondria-dependent pathway mediated by ROS, arresting the cell cycle via PI3K/Akt/mTOR signaling pathway. Hence, our results suggested that ferrocene derivatives F1 and F3 could act as new therapeutic agents against T-ALL.

Apoptosis is a form of normal programmed cell death, which occupies a prominent role in cancer therapy [28]. Generally, the classic apoptosis pathways are divided into extrinsic death and intrinsic mitochondrial death. Numerous studies have shown that the latter is the most common pathway and plays an essential role in the regulation of apoptosis [29,30]. Usually, the Bcl-2 family proteins are involved in the intrinsic mitochondrial apoptosis pathway and the ratio of Bcl-2/Bax is a critical indicator of apoptosis [31]. A decrease in the Bcl-2/Bax index could lead to the loss of MMP and improving the permeability of the mitochondrial membranes, which would allow Cytochrome c from mitochondrial to release into the cytoplasm [32,33]. Subsequently, culminated cytoplasmic Cytochrome c results in the activation of Caspase-9 and Caspase-3, inducing dissociation of PARP [34]. Accordingly, the expression of several related apoptosis factors was investigated first. The data showed that F1 and F3 decreased the expression of Bcl-2, but increased the expression of Bax, Cytochrome c, Cleaved Caspase-9, and Cleaved Caspase-3. These results obtained indicated that a mitochondrial-dependent apoptosis pathway might be involved in Jurkat cells.

Oxidative stress generally plays a crucial role in apoptosis, associated with ROS generation. Previous literature has demonstrated that the pro-apoptotic activity of ferrocene derivatives was associated with ROS production in the Fenton Reaction [35,36]. It may cause depolarization of the mitochondrial membrane when ROS accumulates in the mitochondria, and it is well accepted that a decrease in MMP level could be often used as an indicator of cells undergoing apoptosis [37]. A significant dose-dependent increase in intracellular ROS along with the decrease of MMP was observed in Jurkat cells treated by F1 and F3, which suggested that F1 and F3 induced apoptosis through the mitochondria-dependent pathway mediated by ROS.

On the other hand, effective control of cancer cell proliferation is critical to the prevention of oncogenesis and successful cancer therapy. It is well known that cell cycle progression is tightly linked with cell growth [38]. Previous studies have reported that the cell cycle is regulated by the continuous activation of Cyclin-dependent kinases (CDKs) and Cyclins. Among them, Cyclin D1 and CDK6 play a vital role in the G0/G1 phase, therefore the inhibition of Cyclin D1 and CDK6 could be considered as a promising strategy for the treatment of cancers [39]. Moreover, P21 and P27 act as an inhibitor that has been shown to down-regulate the expression of CDKs, resulting in cell cycle arrest at the G0/G1 phase [40]. It was verified in our study (Figure 5) that the percentage of cells in the G0/G1 phase increased markedly after treated with F1 or F3, and the expression of Cyclin D1 and CDK6 were both decreased while P21 and P27 were increased. Additionally, a Sub-G1 peak indicating DNA damage associated with apoptotic or necrotic processes [41] was observed in Jurkat cells treated by F1 or F3, which was consistent with the apoptosis assays.

The PI3K/Akt/mTOR signal has always been considered as a crucial promoter of cell growth and cell cycle progression, which plays an essential role in the development and progression of cancers [42,43]. It has been confirmed that the inhibition of the PI3K/Akt/mTOR signaling pathway has exhibited encouraging outcomes in various types of cancer, especially hematological malignancies, and might be a potential therapeutic approach for T-ALL [44,45]. In the PI3K/Akt/mTOR signaling pathway, Akt is regarded as a major downstream effector molecule of PI3K, which could cause the activated GSK-3β kinase to prevent the degradation of Cyclin D1 and activation of apoptosis-related factors, thereby accelerating cell cycle development and ultimately promoting cell proliferation [46,47]. In addition, mTOR is viewed as another PI3K/Akt/mTOR signaling pathway downstream component which could also be phosphorylated by Akt [48]. Previous studies reported that mTOR could not only integrate the upstream signals received but also regulate the phosphorylation of p70 S6K and 4E-BP1, thus directly promoting cell growth [49]. The results obtained above supported that both F1 and F3 inhibited the expression of p-Akt (Ser473), p-Akt (Thr308), p-GSK-3β (Ser9), p-mTOR (Ser2448), and p-p70 S6K (Ser389), whereas the expression of Akt, GSK-3β, mTOR, and p70 S6K had no significant change in Jurkat cells. These findings demonstrated that F1 and F3 suppressed the proliferation and cell cycle progression of Jurkat cells through the PI3K/Akt/mTOR signaling pathway.

## 5. Conclusions

In summary, we first disclosed that F1 and F3 displayed significant selectivity for Jurkat cells and low cytotoxicity for normal cells (normal human T and HEK293). Mechanistically, F1 and F3 showed anti-proliferation of Jurkat cells by inducing apoptosis through a mitochondria-dependent pathway mediated by ROS and arresting the cell cycle via the PI3K/Akt/mTOR signaling pathway. Based on these findings, both F1 and F3 could be considered as promising anti-T-ALL drug candidates. Exploring the effect of F1–F7 on other different types of tumor cells and confirming the anti-T-ALL activity of F1 and F3 in vivo is in the process.

## Data Availability

The data presented in this study are available on request from the corresponding author. The data are not publicly available due to due to restrictions privacy or ethical.

## Supplementary Materials

The following are available online at https://www.mdpi.com/article/10.3390/cancers13184677/s1. Table S1: The IC_50_ of compounds F1, F3 and Daunorubicin in three T-ALL cell lines (Jurkat, CEM-T4, Molt-4) and two normal cell lines (Normal human T, HEK293). Figure S1: Chemical structures of the seven novel ferrocene derivatives (compounds F1–F7). Figure S2: The inhibition rate (%) of ferrocene derivatives F1–F7 in Jurkat, CEM-T4, RAJI, CA46, SNT8, and SNK6 cells. F1–F7 at different concentrations (6.25, 12.5, 25, 50 and 100 µM) for 48 h. Figure S3: Detailed information of protein expression analysis by Western blot.

## References

[1] Siegel R.L., Miller K.D., Fuchs H.E., Jemal A. (2021). Cancer statistics, 2021. CA Cancer J. Clin..

[2] Malard F., Mohty M. (2020). Acute lymphoblastic leukaemia. Lancet.

[3] Hong W.H., Cheng W.Z., Zheng T.J., Jiang N., Xu R.A. (2020). AHR is a tunable knob that controls HTLV-1 latency-reactivation switching. PLoS Pathog..

[4] Cheng W.Z., Zheng T.J., Wang Y., Cai K., Wu W.C., Zhao T.J., Xu R.A. (2019). Activation of Notch1 signaling by HTLV-1 Tax promotes proliferation of adult T-cell leukemia cells. Biochem. Biophys. Res. Commun..

[5] Arber D.A., Orazi A., Hasserjian R. (2016). The 2016 revision to the World Health Organization classification of myeloid neoplasms and acute leukemia. Blood.

[6] Marks D.I., Rowntree C. (2017). Management of adults with T-cell lymphoblastic leukemia. Blood.

[7] Pui C.H., Evans W.E. (2013). A 50-Year Journey to Cure Childhood Acute Lymphoblastic Leukemia. Semin. Hematol..

[8] Lu H., Yuan G.X., He Q.H., Chen H.W. (2009). Rapid analysis of anthracycline antibiotics doxorubicin and daunorubicin by microchip capillary electrophoresis. Microchem. J..

[9] Evangelisti C., Chiarini F., McCubrey J.A., Martelli A.M. (2018). Therapeutic Targeting of mTOR in T-Cell Acute Lymphoblastic Leukemia: An Update. Int. J. Mol. Sci..

[10] Patra M., Gasser G. (2017). The medicinal chemistry of ferrocene and its derivatives. Nat. Rev. Chem..

[11] Plazuk D., Top S., Vessieres A., Plamont M.A., Huche M., Zakrzewski J., Makal A., Wozniak K., Jaouen G. (2010). Organometallic cyclic polyphenols derived from 1,2-(alpha-keto tri or tetra methylene) ferrocene show strong antiproliferative activity on hormone-independent breast cancer cells. Dalton Trans..

[12] Boros E., Dyson P.J., Gasser G. (2020). Classification of Metal-Based Drugs according to Their Mechanisms of Action. Chem.

[13] Bruyere C., Mathieu V., Vessieres A., Pigeon P., Top S., Jaouen G., Kiss R. (2014). Ferrocifen derivatives that induce senescence in cancer cells: Selected examples. J. Inorg. Biochem..

[14] Shagufta, Ahmad, I. (2018). Tamoxifen a pioneering drug: An update on the therapeutic potential of tamoxifen derivatives. Eur. J. Med. Chem..

[15] Spencer J., Amin J., Wang M.H. (2011). Synthesis and biological evaluation of JAHAs: Ferrocene-Based Histone Deacetylase Inhibitors. ACS Med. Chem. Lett..

[16] Sun A.J., Lin J.S., Pi C., Xu R.A., Cui X.L. (2016). Biological Evaluation of Ferrocenyl Olefins: Cancer Cell Growth Inhibition, ROS Production, and Apoptosis Activity. Arch. Pharm..

[17] Zheng J.R., Zeng L., Tang M.Q., Lin H.J., Cui X.L. (2021). Novel Ferrocene Derivatives Induce G0/G1 Cell Cycle Arrest and Apoptosis through the Mitochondrial Pathway in Human Hepatocellular Carcinoma. Int. J. Mol. Sci..

[18] Pi C., Cui X.L., Liu X.Y., Guo M.X., Zhang H.Y., Wu Y.J. (2014). Synthesis of Ferrocene Derivatives with Planar Chirality via Palladium-Catalyzed Enantioselective C-H Bond Activation. Org. Lett..

[19] Gioia L., Siddique A., Head S.R., Salomon D.R., Su A.I. (2018). A genome-wide survey of mutations in the Jurkat cell line. BMC Genom..

[20] Ratiani L., Sanikidze T., Sulakvelidze M., Bejitashvili N., Meladze K. (2009). Jurkat cell as an appropriate model for drug investigation. Georgian Med. News.

[21] Kowalski K., Hikisz P., Szczupak L., Therrien B., Koceva-Chyla A. (2014). Ferrocenyl and dicobalt hexacarbonyl chromones-New organometallics inducing oxidative stress and arresting human cancer cells in G2/M phase. Eur. J. Med. Chem..

[22] Novgorodov S.A., Voltin J.R., Gooz M.A., Li L., Lemasters J.J., Gudz T.I. (2018). Acid sphingomyelinase promotes mitochondrial dysfunction due to glutamate-induced regulated necrosis. J. Lipid Res..

[23] Cassidy-Stone A., Chipuk J., Ingerman E. (2008). Chemical inhibition of the mitochondrial division dynamin reveals its role in Bax/Bak-dependent mitochondrial outer membrane permeabilization. Dev. Cell.

[24] Lopez J., Tait S. (2015). Mitochondrial apoptosis: Killing cancer using the enemy within. Brit. J. Cancer.

[25] Aoki M., Fujishita T. (2017). Oncogenic Roles of the PI3K/AKT/mTOR Axis. Curr. Top. Microbiol..

[26] Buttrick G., Wakefield J. (2008). PI3K and GSK-3: Akting together with microtubules. Cell Cycle.

[27] Shams H.Z., Mohareb R.M., Helal M.H. (2011). Design and Synthesis of Novel Antimicrobial Acyclic and Heterocyclic Dyes and Their Precursors for Dyeing and/or Textile Finishing Based on 2-N-Acylamino-4,5,6,7-tetrahydrobenzo [b] thiophene Systems. Molecules.

[28] Elmore S. (2007). Apoptosis: A review of programmed cell death. Toxicol. Pathol..

[29] Lam M., Oleinick N.L., Nieminen A.L. (2001). Photodynamic therapy-induced apoptosis in epidermoid carcinoma cells-Reactive oxygen species and mitochondrial inner membrane permeabilization. J. Biol. Chem..

[30] Shi M.C., Zhou L.N., Zhao L., Shang M., He T.T., Tang Z.L., Sun H.C., Ren P.L., Lin Z.P., Chen T.J. (2017). Csseverin inhibits apoptosis through mitochondria-mediated pathways triggered by Ca^2+^ dyshomeostasis in hepatocarcinoma PLC cells. PLoS Negl. Trop. Dis..

[31] Xu W.C., Wang X.Q., Tu Y.C., Masaki H., Tanaka S., Onda K., Sugiyama K., Yamada H., Hirano T. (2019). Tetrandrine and cepharanthine induce apoptosis through caspase cascade regulation, cell cycle arrest, MAPK activation and PI3K /Akt / mTOR signal modification in glucocorticoid resistant human leukemia Jurkat T cells. Chem.-Biol. Interact..

[32] Soriano M.E., Scorrano L. (2011). Traveling Bax and forth from mitochondria to control apoptosis. Cell.

[33] Li P., Nijhawan D., Budihardjo I., Srinivasula S.M., Ahmad M., Alnemri E.S., Wang X. (1997). Cytochrome c and dATP-dependent formation of Apaf-1/caspase-9 complex initiates an apoptotic protease cascade. Cell.

[34] Koh D.W., Dawson T.M., Dawson V.L. (2005). Mediation of cell death by poly (ADP-ribose) polymerase-1. Pharmacol. Res..

[35] Dixon S.J., Stockwell B.R. (2014). The role of iron and reactive oxygen species in cell death. Nat. Chem. Biol..

[36] Huang H., Chen J., Lu H., Zhou M., Chai Z., Hu Y. (2017). Iron-induced generation of mitochondrial ROS depends on AMPK activity. Biometals.

[37] Reed J.C., Pellecchia M. (2005). Review in translational hematology Apoptosis-based therapies for hematologic malignancies. Cell.

[38] Adjei A. (2001). Blocking Oncogenic Ras signaling for Cancer Therapy. J. Natl. Cancer Inst..

[39] Malumbres M. (2014). Cyclin-dependent kinases. Genome Biol..

[40] Starostina N.G., Kipreos E.T. (2012). Multiple degradation pathways regulate versatile CIP/KIP CDK inhibitors. Trends Cell Biol..

[41] Riccardi C., Nicoletti I. (2006). Analysis of apoptosis by propidium iodide staining and flow cytometry. Nat. Protoc..

[42] Zhou X., Yang Y. (2019). TRIM44 is indispensable for glioma cell proliferation and cell cycle progression through AKT/p21/p27 signaling pathway. J. Neuro-Oncol..

[43] Laplante M., Sabatini D.M. (2012). mTOR signaling in growth control and disease. Cell.

[44] Bongiovanni D., Saccomani V., Piovan E. (2017). Aberrant signaling pathways in T-cell acute lymphoblastic leukemia. Int. J. Mol. Sci..

[45] Oliveira M., Akkapeddi P. (2017). From the outside, from within: Biological and therapeutic relevance of signal transduction in T-cell acute lymphoblastic leukemia. Cell. Signal..

[46] Bowles D., Jimeno A. (2011). New phosphatidylinositol 3-kinase inhibitors for cancer. Expert Opin. Investig. Drugs.

[47] Baer R., Cintas C., Dufresne M. (2014). Pancreatic cell plasticity and cancer initiation induced by oncogenic Kras is completely dependent on wild-type PI 3-kinase p110α Genes. Gene. Dev..

[48] Bhat M., Robichaud N., Hulea L. (2015). Targeting the translation machinery in cancer. Nat. Rev. Drug Discov..

[49] Ben-Sahra I., Hoxhaj G., Ricoult S.J.H. (2016). mTORC1 induces purine synthesis through control of the mitochondrial tetrahydrofolate cycle. Science.

