# Supplementary material for: Novel Ferrocene Derivatives Induce Apoptosis through Mitochondria-Dependent and Cell Cycle Arrest via PI3K/Akt/mTOR Signaling Pathway in T Cell Acute Lymphoblastic Leukemia
Source: Cancers (Basel). 2021 Sep 18;13(18):4677. doi: 10.3390/cancers13184677 (PMC8467123; doi:10.3390/cancers13184677)
Supplement: Supplementary file 1 [file cancers-13-04677-s001.zip › cancers-1366270-supplementary.pdf]

Supplementary Materials: Novel Ferrocene Derivatives Induce Apoptosis through Mitochondria-Dependent and Cell Cycle Arrest via PI3K/Akt/mTOR Signaling Pathway in T Cell Acute Lymphoblastic Leukemia

Liao Zeng, Mingqing Tang, Chao Pi, Jianrong Zheng, Sanxing Gao, Titaua Chabanne, Remi Chauvin, Wenzhao Cheng, Hongjun Lin, Ruian Xu and Xiuling Cui

Table S1. The IC_{50} of compound F1, F3, and Daunorubicin in three T-ALL cell lines (Jurkat, CEM-T4, Molt-4) and normal cell lines (Normal human T and HEK293).

Compounds/ IC50 (μM)	Jurkat	CEM-T4	Molt-4	Normal Human T	HEK293
F1	18.17 ± 1.23	30.77 ± 2.80	28.22 ± 1.17	190.03 ± 5.78	127.25 ± 2.60
F3	27.73 ± 0.97	44.43 ± 1.50	43.56 ± 1.21	242.59 ± 3.34	135.60 ± 1.70
Daunorubicin	0.08 ± 0.01	0.06 ± 0.01	0.05 ± 0.01	0.12± 0.01	0.02 ± 0.01

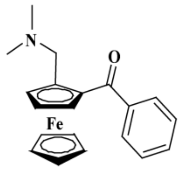
F1

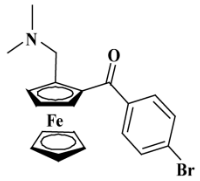
F2

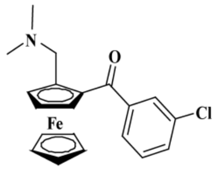
F3

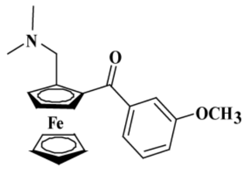
F4

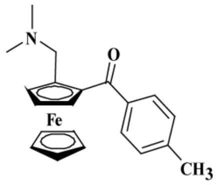
F5

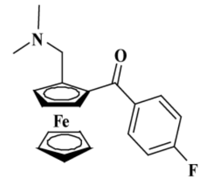
F6

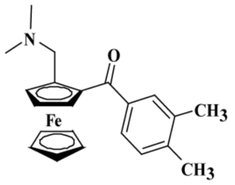
F7

Figure S1. Chemical structures of the seven novel ferrocene derivatives (compounds F1–F7).

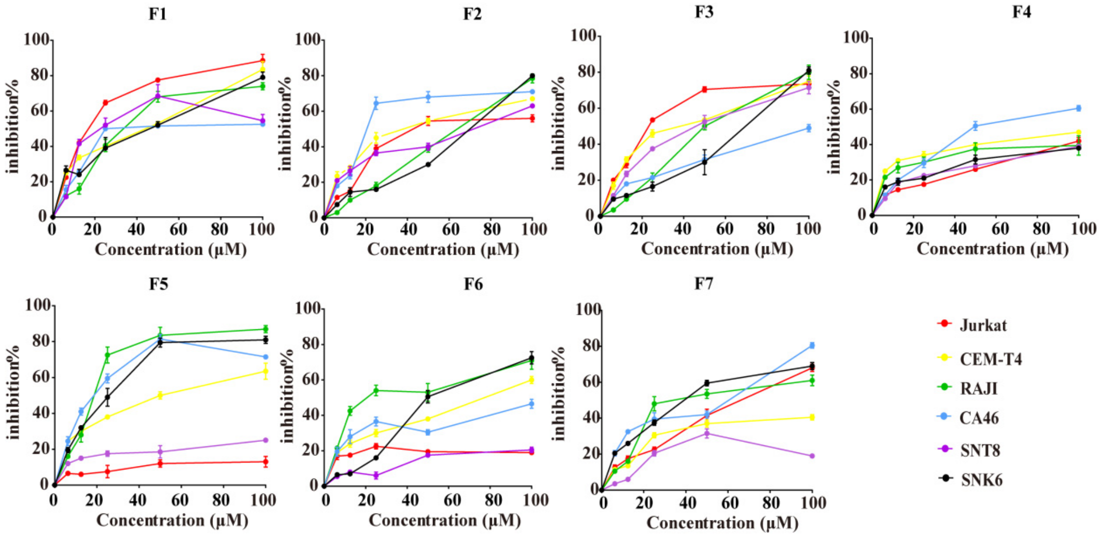

Figure S2. The inhibition rate (%) of ferrocene derivatives F1–F7 in Jurkat, CEM-T4, RAJI, CA46, SNT8, and SNK6 cells. F1–F7 at different concentrations (6.25, 12.5, 25, 50 and 100 μM) for 48 h. The data were expressed as the mean ± SEM of three independent experiments.

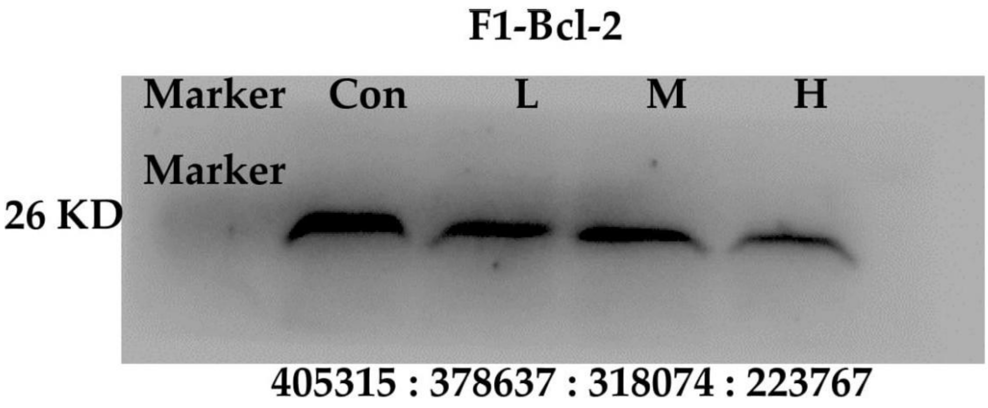

(correspondence to Figure 4A in the manuscript)

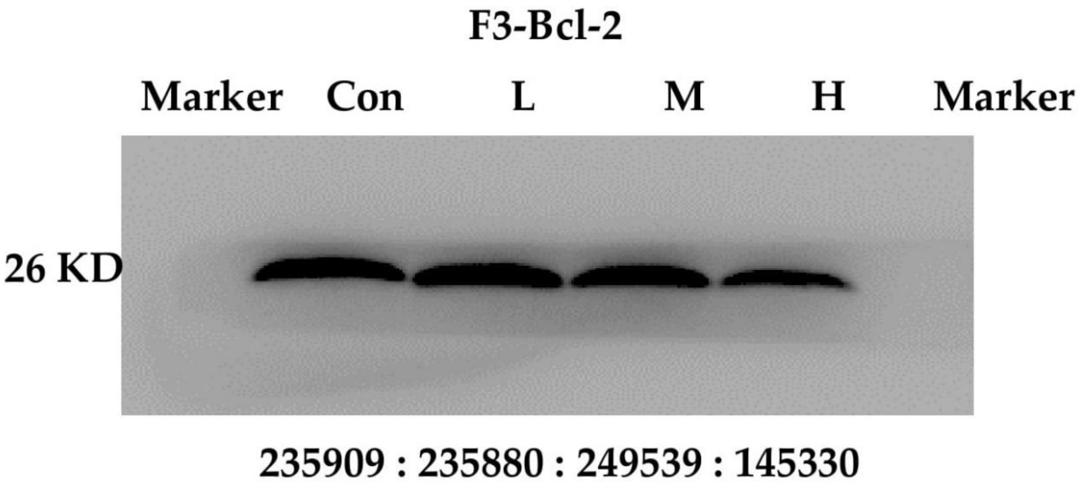

(correspondence to Figure 4A in the manuscript)

F1-Bax

Marker Con L M H Marker

20 KD

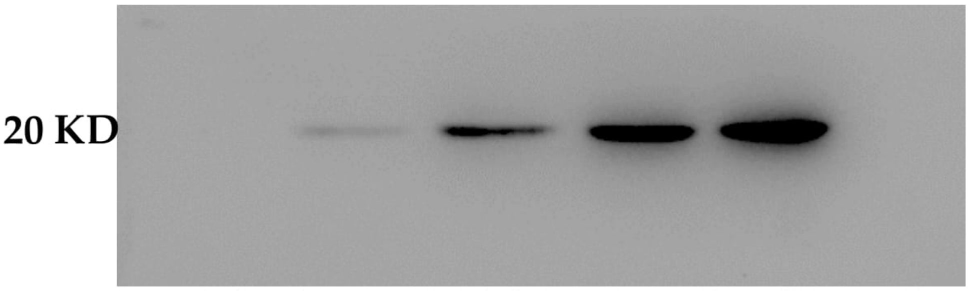

27991 : 168598 : 310678 : 416468

(correspondence to Figure 4A in the manuscript)

F3-Bax

Marker Con L M H Marker

20 KD

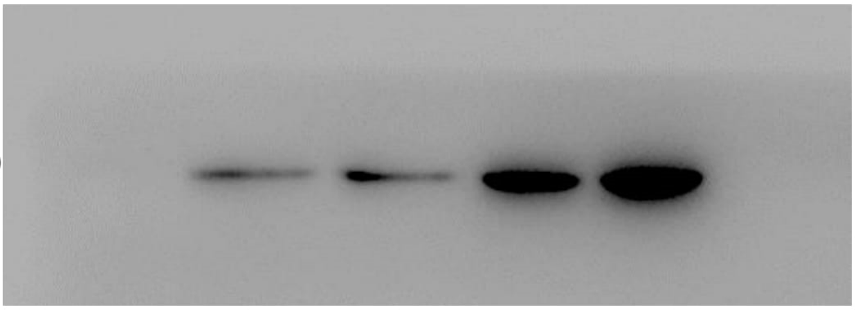

134375 : 169679 : 343267 : 470405

(correspondence to Figure 4A in the manuscript)

F1-Cyto c

Marker Con L M H Marker

14 KD

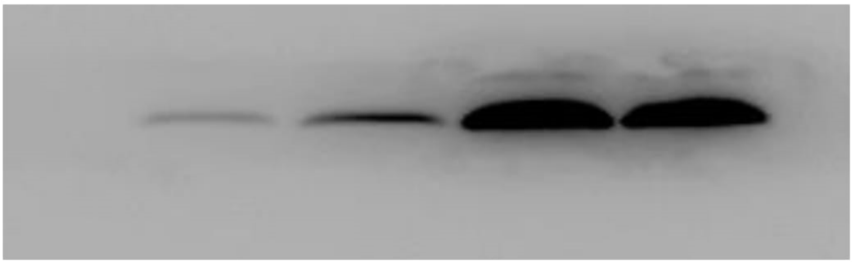

45832 : 130562 : 342213 : 309247

(correspondence to Figure 4A in the manuscript)

F3-Cyto c

Marker Con L M H Marker

14 KD

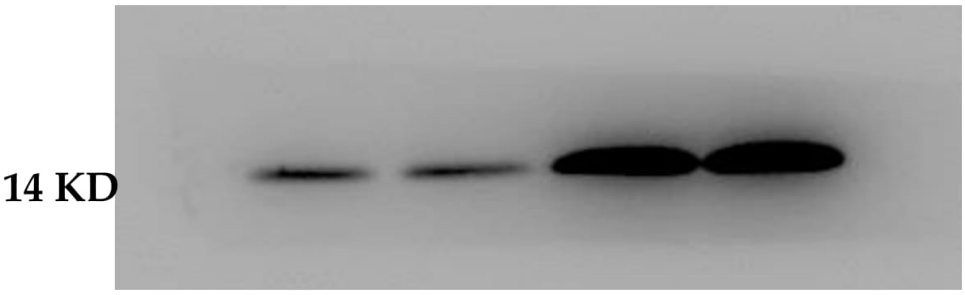

89660 : 92724 : 269171 : 309731

(correspondence to Figure 4A in the manuscript)

F1-Cleaved Caspase-9

Marker Con L M H Marker

37 KD

35 KD

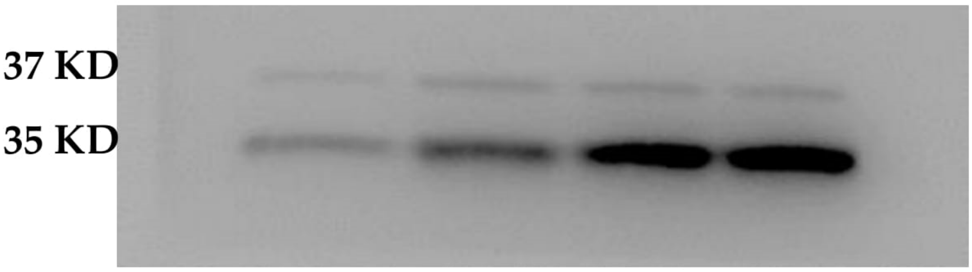

9252: 21170: 28051: 25617

48694: 125568: 185202: 20586

(correspondence to Figure 4A in the manuscript)

F3-Cleaved Caspase-9

Marker Con L M H Marker

37 KD

35 KD

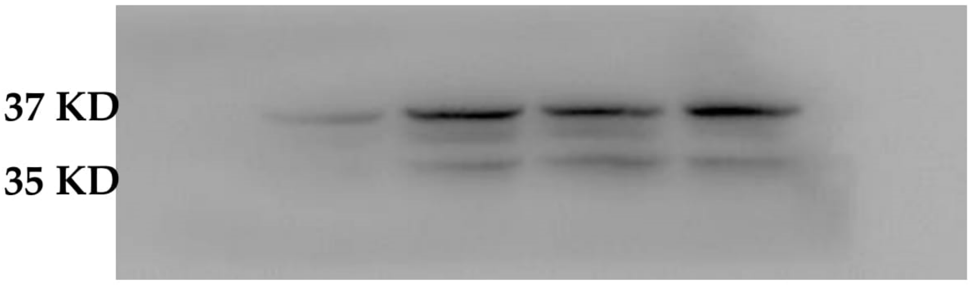

32852: 120505: 137657: 129479

8712 : 40651: 61516: 58122

(correspondence to Figure 4A in the manuscript)

F1-Cleaved Caspase-3

Marker Con L M H

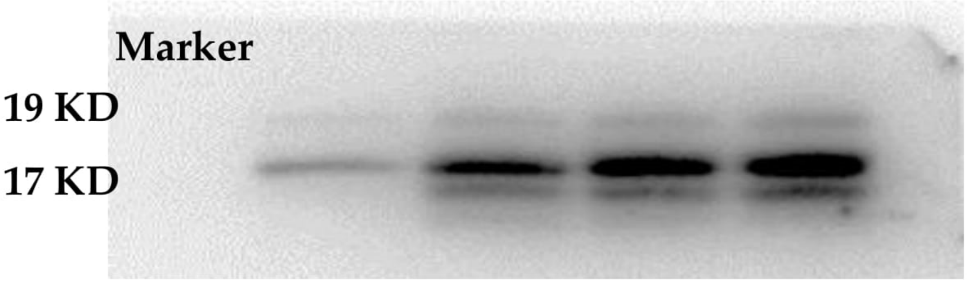

36374: 140296: 163125: 161751

5336: 56754: 72791: 98672

(correspondence to Figure 4A in the manuscript)

F3-Cleaved Caspase-3

Marker Con L M H Marker

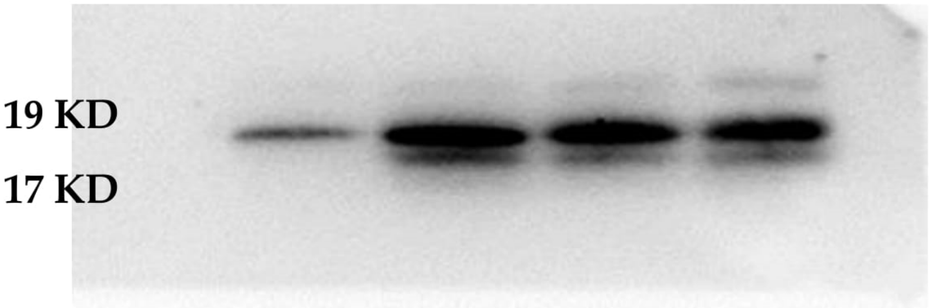

55347: 150005: 153878: 124664

12456: 65943: 52121: 56948

(correspondence to Figure 4A in the manuscript)

F1-PARP

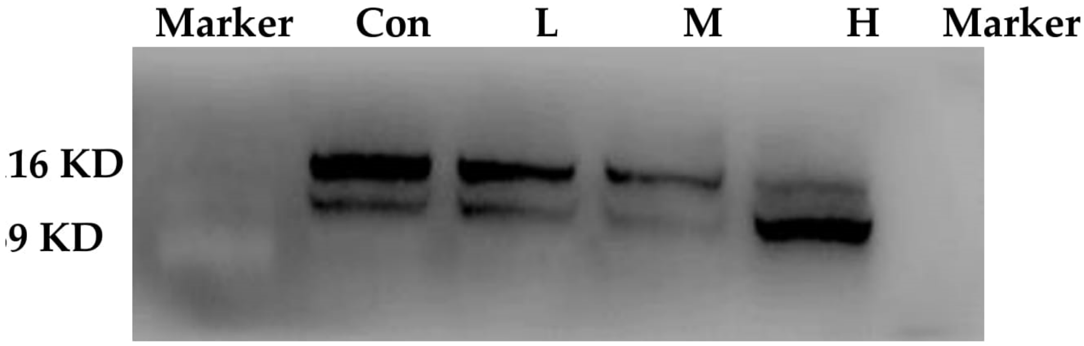

205394: 192457: 163305: 127396

160426: 166331: 127465: 237972

(correspondence to Figure 4A in the manuscript)

F3-PARP

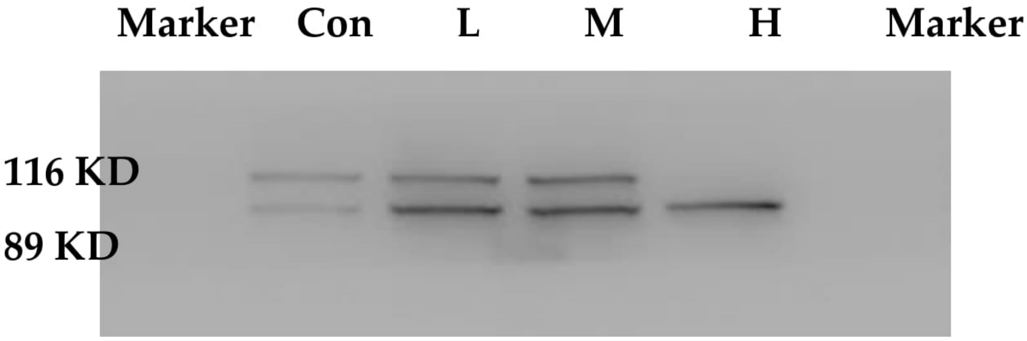

120789:126129:116658: 53144

68970: 65600: 94679: 90541

(correspondence to Figure 4A in the manuscript)

F1-β-tubulin

Marker Con L M H Marker

50 KD

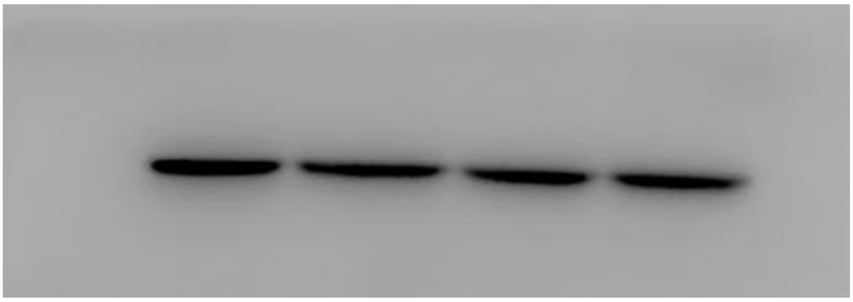

239272 : 191869 : 186471 : 180623

(correspondence to Figure 4A in the manuscript)

F3-β-tubulin

Marker Con L M H Marker

50 KD

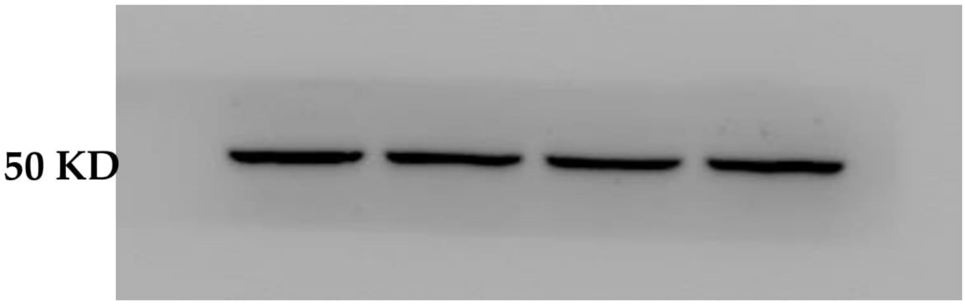

181488 : 180377 : 198710 : 175777

(correspondence to Figure 4A in the manuscript)

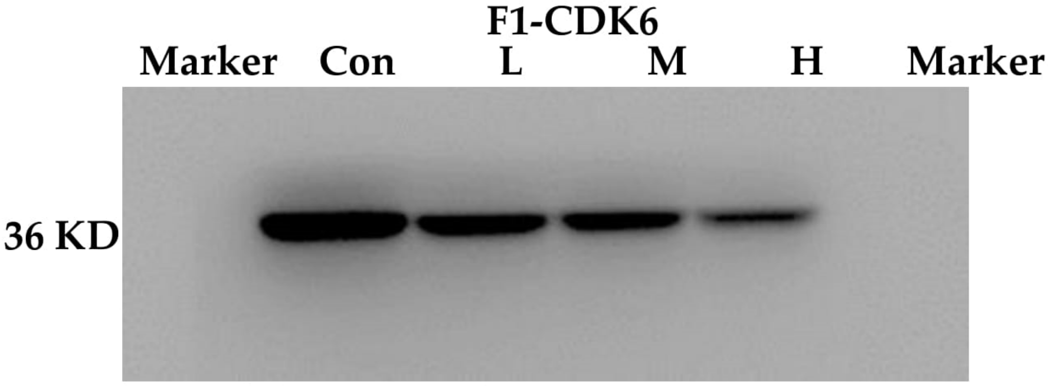

257572 : 198515 : 140945 : 85799

(correspondence to Figure 5A in the manuscript)

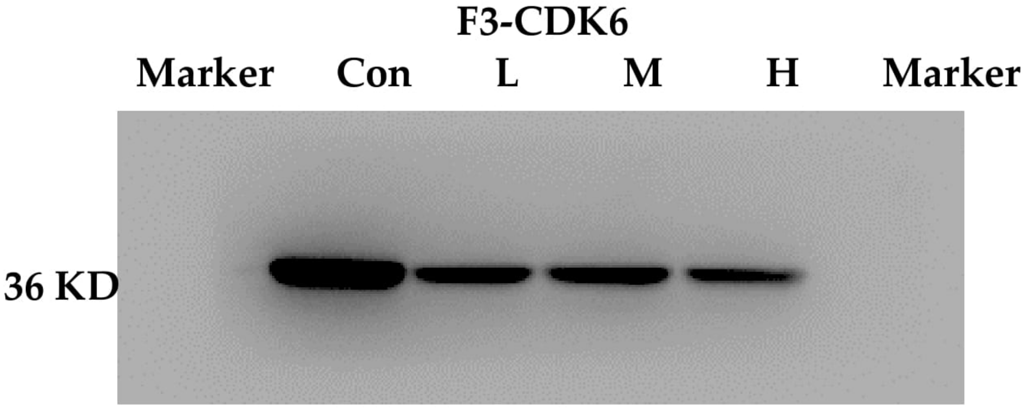

332098 : 163091 : 194267 : 149327

(correspondence to Figure 5A in the manuscript)

F1-Cyclin D1

Marker Con L M H Marker

36 KD

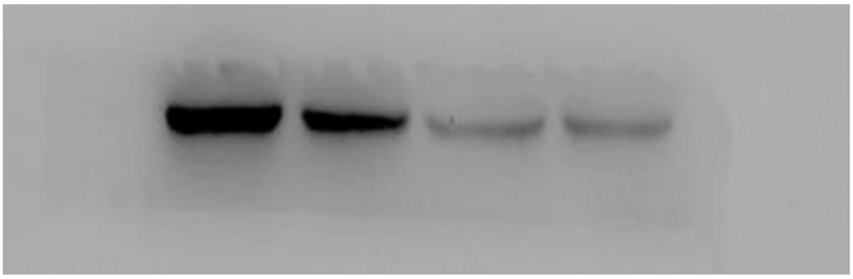

341062:246324:134855:122808

(correspondence to Figure 5A in the manuscript)

F3-Cyclin D1

Marker Con L M H Marker

36 KD

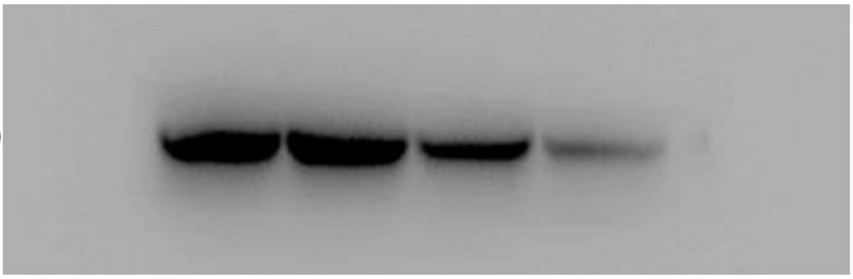

381371:368293:268948:159010

(correspondence to Figure 5A in the manuscript)

F1-P21

Marker Con L M H Marker

21 KD

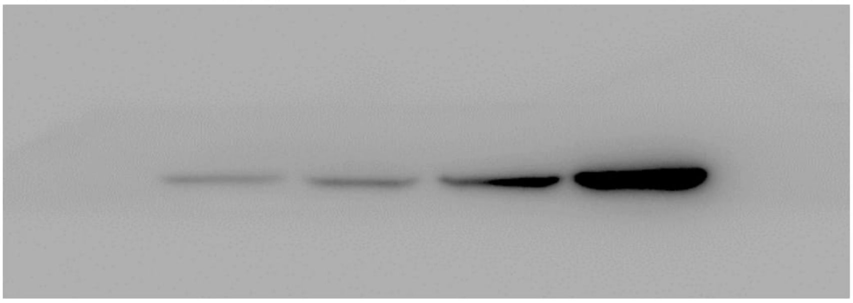

71009 : 86217 : 219540 : 525780

(correspondence to Figure 5A in the manuscript)

F3-P21

Marker Con L M H Marker

21 KD

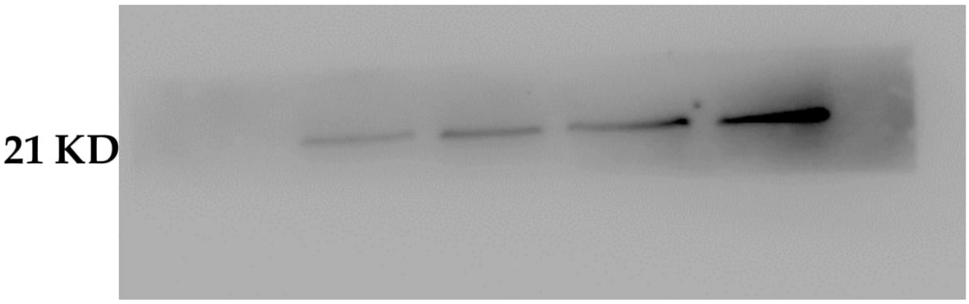

93175 : 101590 : 166586 : 370573

(correspondence to Figure 5A in the manuscript)

F1-P27

Marker Con L M H Marker

27 KD

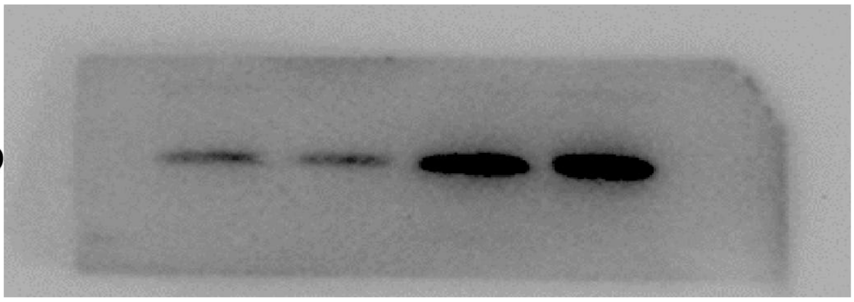

41815 : 46107 : 142581 : 191935

(correspondence to Figure 5A in the manuscript)

F3-P27

Marker Con L M H Marker

27 KD

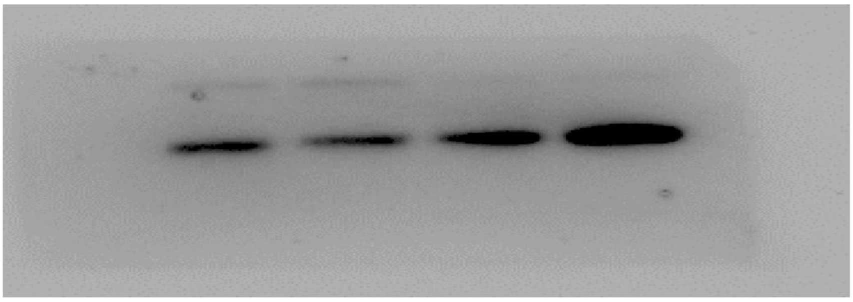

124354 : 121587 : 178561 : 286274

(correspondence to Figure 5A in the manuscript)

F1-β-tubulin

Marker Con L M H Marker

50 KD

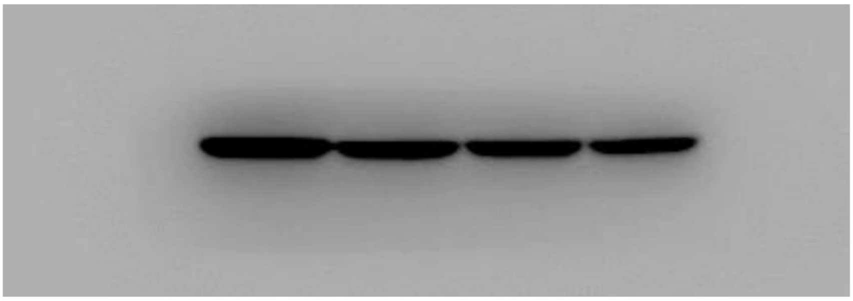

241927 : 211265 : 191013 : 183527

(correspondence to Figure 5A in the manuscript)

F3-β-tubulin

Marker Con L M H Marker

50 KD

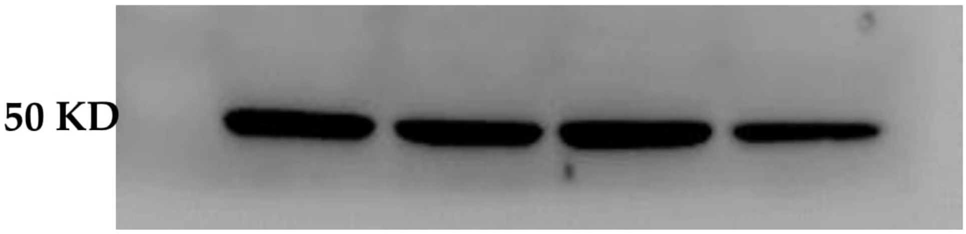

211959 : 222096 : 248876 : 194520

(correspondence to Figure 5A in the manuscript)

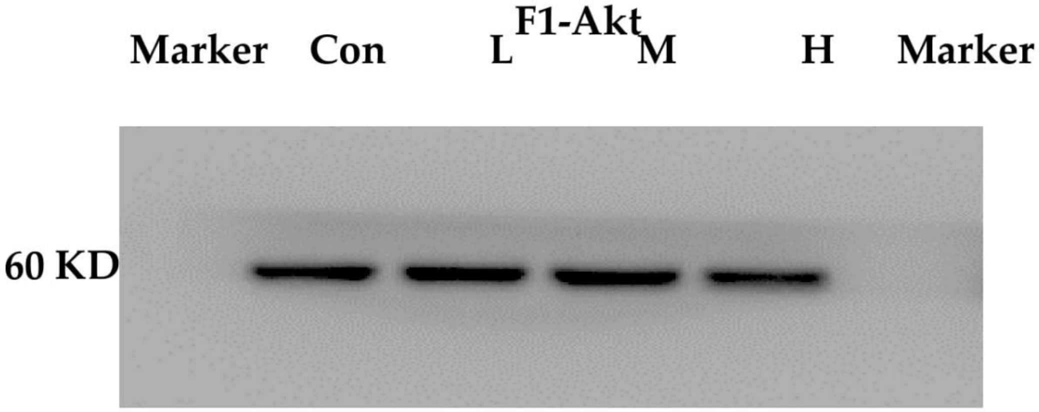

138734 : 139920 : 158632 : 140324

(correspondence to Figure 6A in the manuscript)

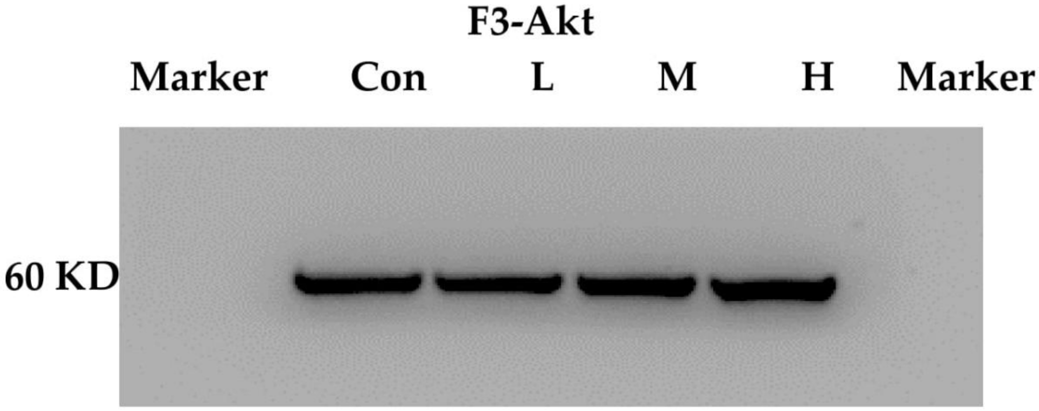

180327 : 154755 : 164418 : 173871

(correspondence to Figure 6A in the manuscript)

F1-p-Akt (Thr308)

Marker Con L M H Marker

60 KD

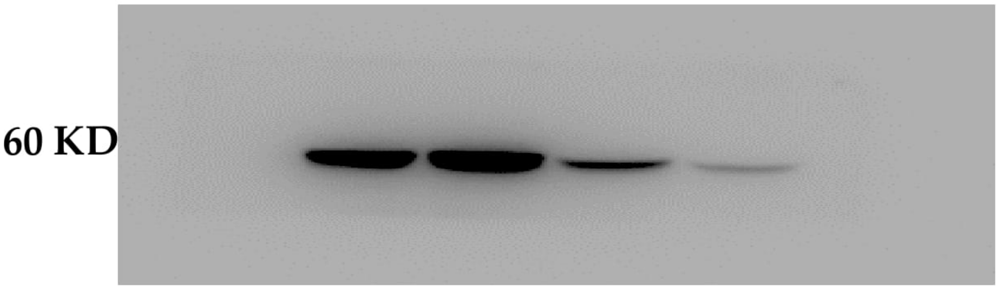

267411 : 298654 : 135245 : 33643

(correspondence to Figure 6A in the manuscript)

F3-p-Akt (Thr308)

Marker Con L M H Marker

60 KD

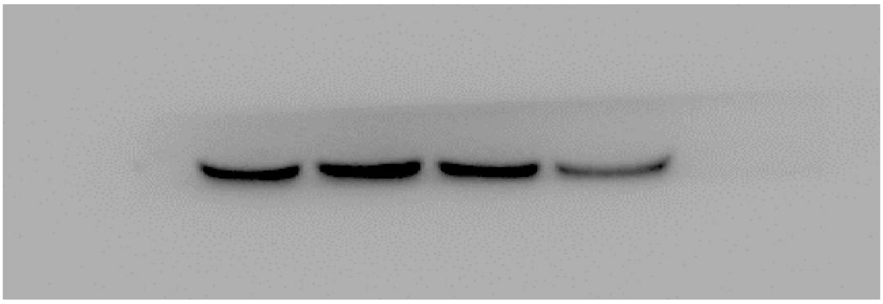

156848 : 167713 : 133136 : 96563

(correspondence to Figure 6A in the manuscript)

F1-p-Akt (Ser473)

Marker Con L M H Marker

60 KD

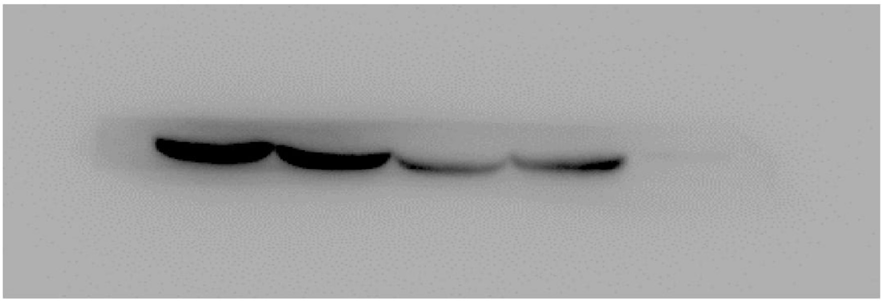

312348 : 246644 : 123789 : 134457

(correspondence to Figure 6A in the manuscript)

F3-p-Akt (Ser473)

Marker Con L M H Marker

[illegible]0 KD

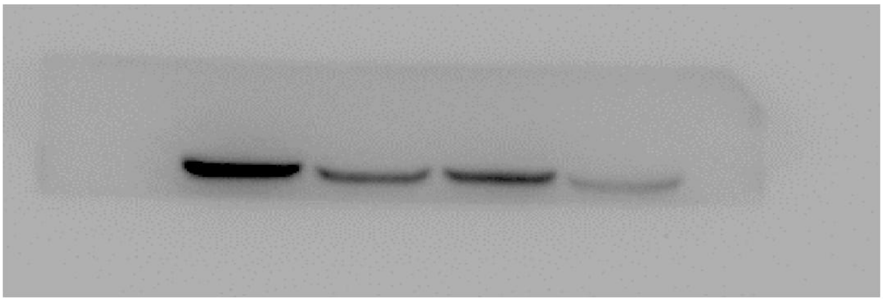

168814 : 77484 : 87714 : 36044

(correspondence to Figure 6A in the manuscript)

F1-GSK-3β

Marker Con L M H Marker

46 KD

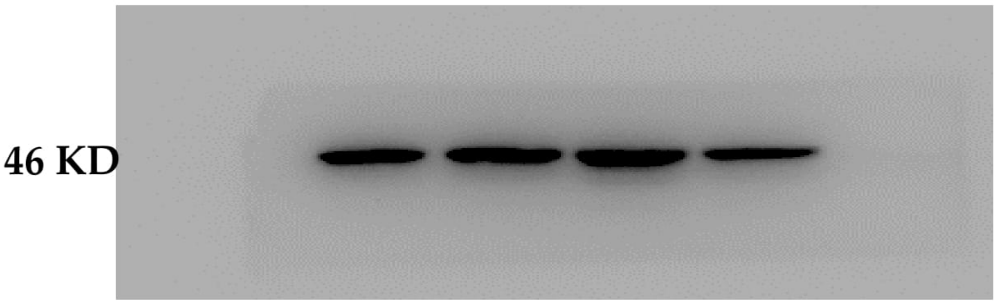

179543 : 157973 : 190961: 157262

(correspondence to Figure 6A in the manuscript)

F3-GSK-3β

Marker Con L M H Marker

46 KD

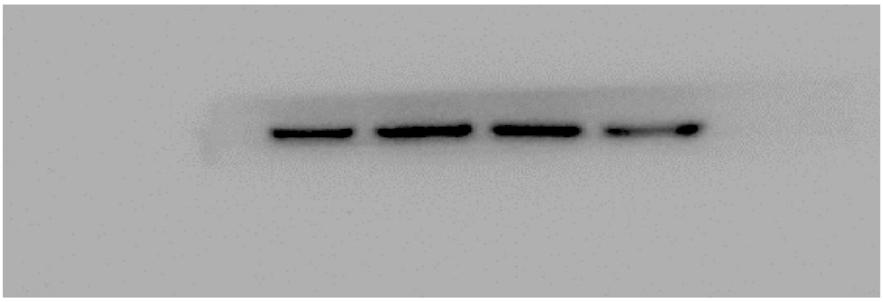

145560 : 165985 : 160288 : 134006

(correspondence to Figure 6A in the manuscript)

F1-p-GSK-3β (Ser9)

Marker Con L M H

46 KD

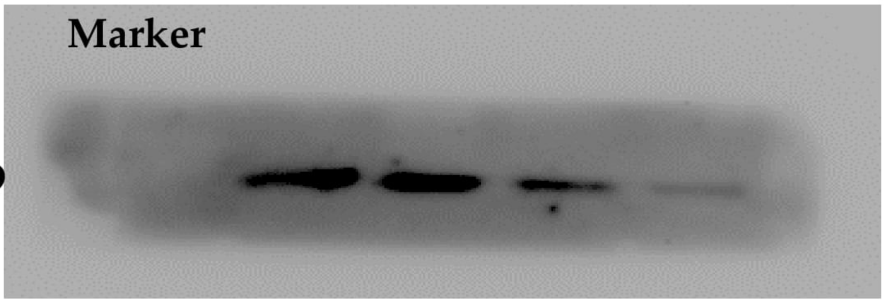

206798 : 122201 : 76942 : 58313

(correspondence to Figure 6A in the manuscript)

F3-p-GSK-3β (Ser9)

Marker Con L M H Marker

46 KD

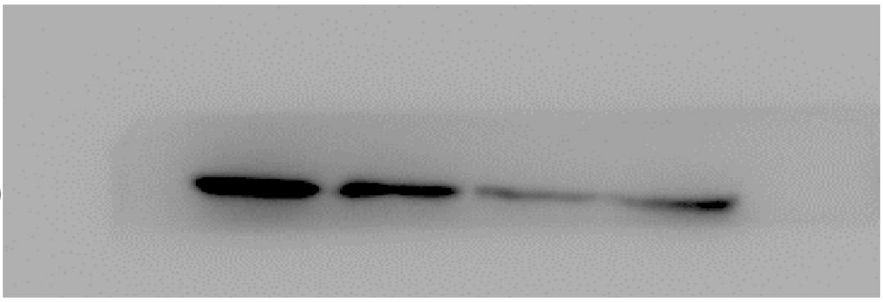

268496 : 192005 : 75913 : 100175

(correspondence to Figure 6A in the manuscript)

F1-mTOR

Marker Con L M H Marker

289 KD

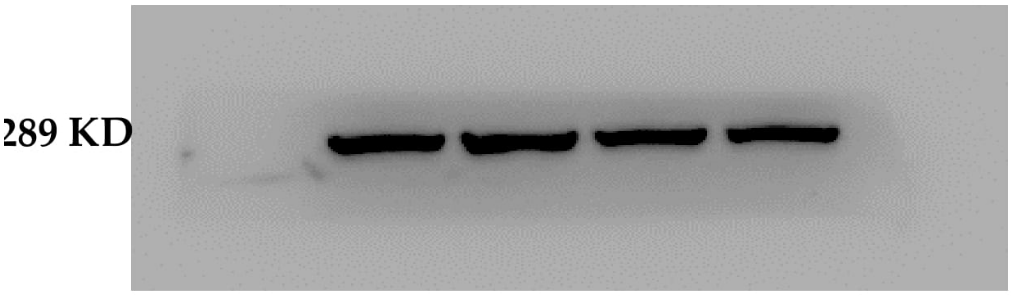

201674 : 210486 : 181499 : 173692

(correspondence to Figure 6A in the manuscript)

F3-mTOR

Marker Con L M H Marker

289 KD

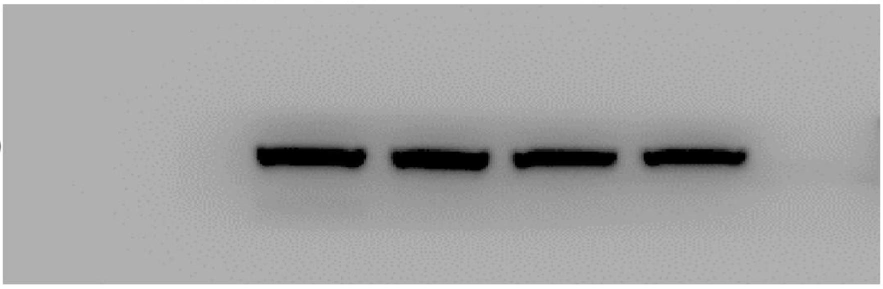

203392 : 181078 : 186718 : 174102

(correspondence to Figure 6A in the manuscript)

F1-p-mTOR (Ser2448)

Marker Con L M H Marker

289 KD

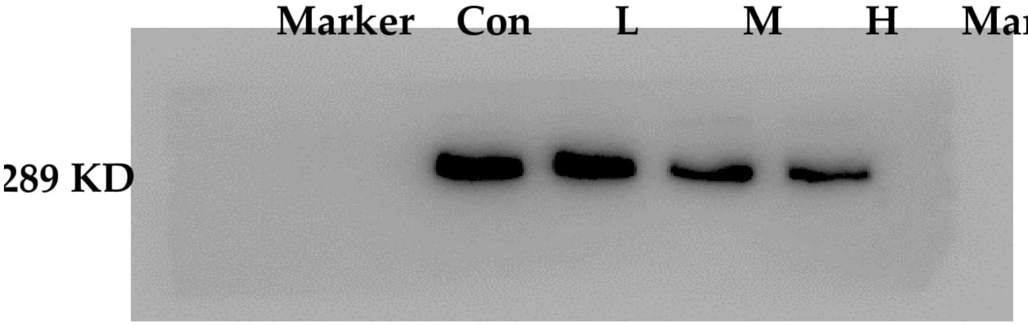

245940 : 235718 : 163981 : 138698

(correspondence to Figure 6A in the manuscript)

F3-p-mTOR (Ser2448)

Marker Con L M H Marker

289 KD

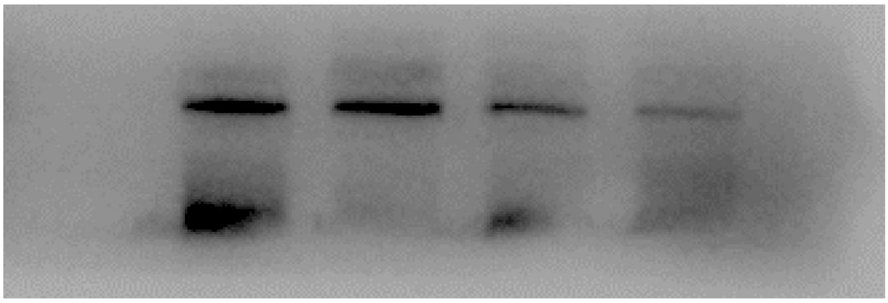

102890 : 73647 : 60219 : 44887

(correspondence to Figure 6A in the manuscript)

F1-p70 S6K

Marker Con L M H Marker

70 KD

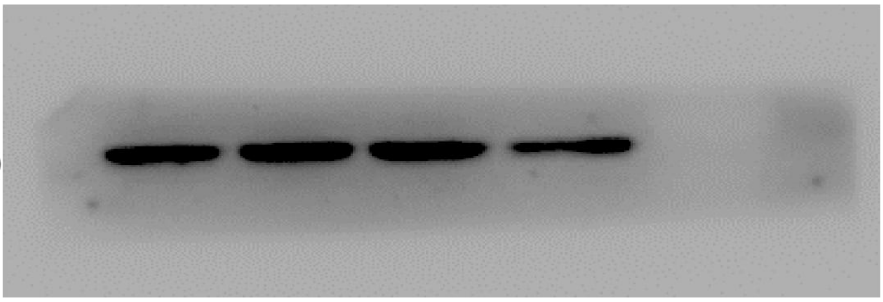

206628 : 192554 : 191760 : 176193

(correspondence to Figure 6A in the manuscript)

F3-p70 S6K

Marker Con L M H Marker

70 KD

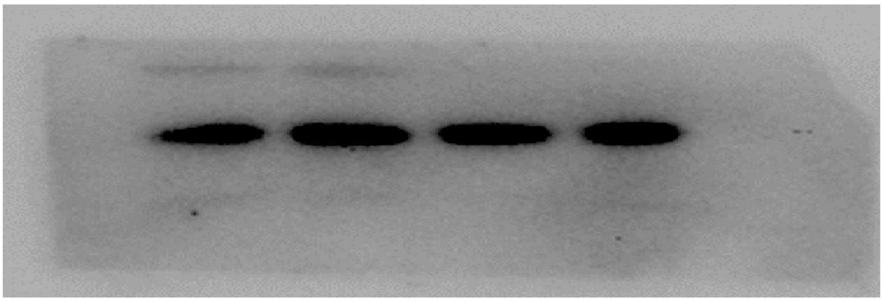

180636 : 226475 : 202978 : 181401

(correspondence to Figure 6A in the manuscript)

F1-p-p70 S6K (Ser389)

Marker Con L M H Marker

70

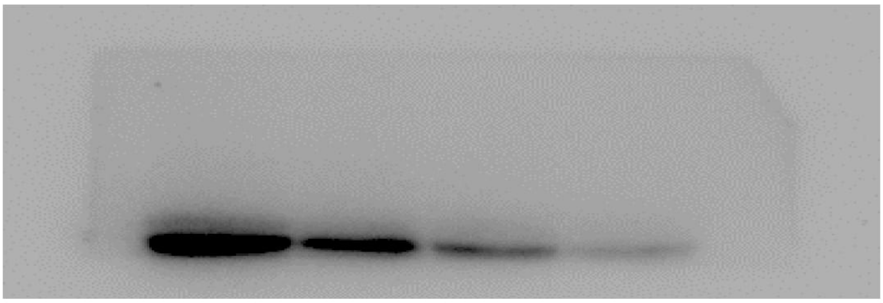

306077 : 166164 : 88709 : 35219

(correspondence to Figure 6A in the manuscript)

F3-p-p70 S6K (Ser389)

Marker Con L M H Marker

70 KD

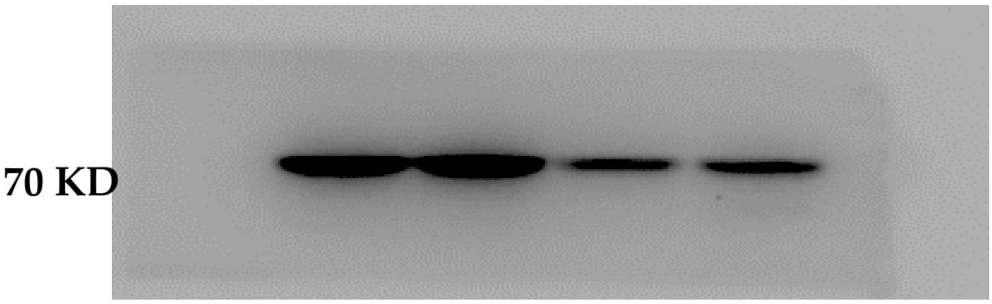

180387 : 191867 : 94395 : 116945

(correspondence to Figure 6A in the manuscript)

F1-GAPDH

Marker Con L M H

36 KD

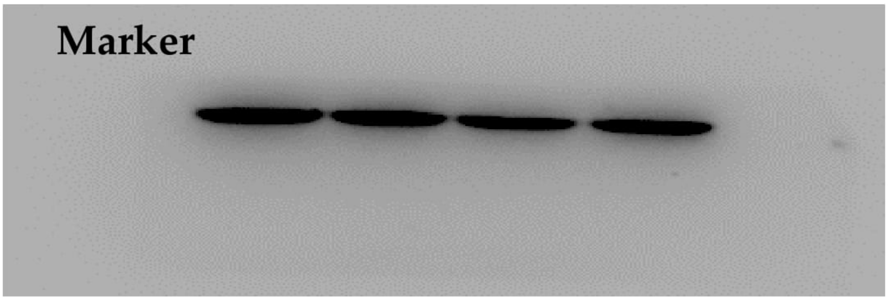

227084 : 208304 : 185087 : 210380

(correspondence to Figure 6A in the manuscript)

F3-GAPDH

Marker Con L M H

36 KD

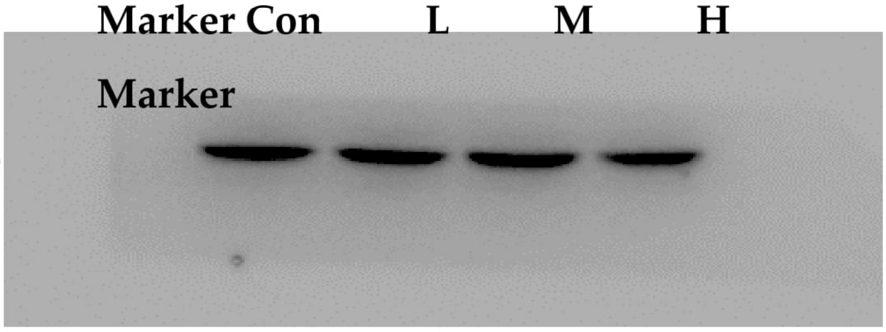

171067 : 166928 : 156244 : 121406

(correspondence to Figure 6A in the manuscript)

Figure S3. Detailed information of protein expression analysis by Western blot.
